# Early Vitamin A Supplementation for Prevention of Short-Term Morbidity and Mortality in Very-Low-Birth-Weight Infants: A Systematic Review and Meta-Analysis

**DOI:** 10.3389/fped.2022.788409

**Published:** 2022-04-07

**Authors:** Yanxiu Ye, Xiaoyan Yang, Jing Zhao, Jianghua He, Xiaoming Xu, Jiao Li, Jing Shi, Dezhi Mu

**Affiliations:** ^1^Department of Pediatrics, West China Second University Hospital, Sichuan University, Chengdu, China; ^2^Key Laboratory of Birth Defects and Related Diseases of Women and Children (Sichuan University), Ministry of Education, Sichuan University, Chengdu, China

**Keywords:** vitamin A, very-low-birth-weight, bronchopulmonary dysplasia, mortality, meta-analysis

## Abstract

**Background:**

Vitamin A plays an important role in the development and maintenance of the normal function of organs and systems. Premature infants have low levels of vitamin A, which may be associated with an increased risk of developing disease. This study aimed to evaluate the effects of vitamin A supplementation on short-term morbidity and mortality in very-low-birth-weight (VLBW) infants.

**Methods:**

We used PubMed, EMBASE, the Cochrane Central Register of Controlled Trials, and Web of Science to conduct a literature search of studies published before January 1, 2022, to be included in our meta-analysis. The analysis included randomized controlled trials that compared the effects of vitamin A supplementation on VLBW infants (birth weight <1,500 g) and controls given a placebo or no treatment. The certainty of evidence was assessed using Grading of Recommendations, Assessment, Development and Evaluation (GRADE) guidelines.

**Results:**

Twelve randomized controlled trials were included in the meta-analysis, and 2,111 infants were pooled and analyzed. The overall risk of bias was not serious in the included studies. Vitamin A supplementation for reducing the incidence of bronchopulmonary dysplasia (BPD) at 36 weeks' postmenstrual age seems to be limited [risk ratio (RR):0.85; 95% confidence intervals (CI): 0.70–1.04; 8 studies, 1,595 infants, very-low-certainty evidence], which is different from the previous systematic review. Length of hospital stay (mean difference: −12.67, 95% CI: −23.55 to −1.79; 6 studies, 739 infants, low-certainty evidence), and the incidence of vitamin A deficiency at 28 days postnatal age (RR: 0.08; 95% CI: 0.02–0.38; 3 studies, 358 infants, low-certainty evidence) were reduced in the vitamin A group. Besides, vitamin A supplementation seems to reduce the incidence of periventricular leukomalacia (RR: 0.68; 95% CI: 0.47–0.97; 4 studies, 1,224 infants, low-certainty evidence) and retinopathy of prematurity of any grade (RR: 0.61; 95% CI: 0.48–0.76; 4 studies, 463 infants, moderate-certainty evidence).

**Conclusions:**

There is no sufficient evidence regarding vitamin A supplementation preventing BPD in VLBW infants. Vitamin A supplementation can reduce the incidence of vitamin A deficiency and retinopathy of prematurity of any grade, and may exert an effect of preventing periventricular leukomalacia.

**Systematic Review Registration:**

http://www.crd.york.ac.uk/PROSPERO/, identifier: CRD42020211070.

## Background

Prematurity is the leading cause of death and disability in children under 5 years of age globally (1). Insufficient nutritional reserves and limited capacity for digestion and absorption in preterm infants often lead to nutrient deficiency. Vitamin A is an essential fat-soluble nutrient, which plays an important role in the development and maintenance of normal function of organs such as the brain, lungs and retina, as well as the immune, digestive, and haematopoietic systems. Humans cannot synthesize vitamin A; thus, it must be obtained through diet. As a result, vitamin A deficiency (VAD) is a major public health problem and the leading cause of blindness in young children (2). The World Health Organization report shows that VAD, along with protein malnutrition, constitute the most common nutritional disorders in the world (3). VAD is common in premature infants because the fetus normally accumulates most of its vitamin A requirements during the third trimester of pregnancy through placental transport, and it takes several months of adequate intake after birth to establish an adequate liver reserve (4). Most premature infants are born with insufficient hepatic vitamin A stores and low serum concentration compared with full-term infants (5, 6). Insufficient supply and malabsorption after birth increases the risk of VAD in preterm infants.

Studies have shown that VAD is associated with an increased risk of BPD (7), retinopathy of prematurity (ROP) (8), infection (2) and delayed neurological development (9). Vitamin A supplementation in children aged 6 months to 5 years who are at risk of VAD has been shown to reduce the risk of all-cause mortality (10). Previous studies have indicated that early supplementation of vitamin A could prevent BPD in VLBW infants and showed a trend toward a lower incidence of ROP and nosocomial sepsis (11). However, in most studies, vitamin A was administered by intramuscular injection, which is invasive and painful for neonates, and its widespread acceptance in clinic practice has been difficult to achieve. Hence, some researchers have conducted clinical trials of oral vitamin A supplementation among preterm infants (12–15). The first of these trials, published in 2001, was for extremely-low-birth-weight (ELBW) infants and showed no benefit from enterally administered vitamin A for the prevention of BPD. Recently other clinical trials of oral vitamin A supplementation showed mixed results regarding its effects on the prevention of BPD and other complications related to prematurity.

A Cochrane meta-analysis published in 2016 showed that early vitamin A supplementation among VLBW infants could reduce the risk of death or oxygen requirement at 1 month of age, as well as the risk of oxygen requirement at 36 weeks' postmenstrual age (PMA). Subsequent studies and systematic reviews indicated that vitamin A supplementation could decrease the risk of ROP (16) and length of hospital stay (17). Previous meta-analyses mainly focused on vitamin A supplementation for BPD prevention. In this meta-analysis, we searched the literature using predefined criteria to determine the effects of vitamin A supplementation on short-term morbidity and mortality in VLBW infants, with the goal of updating previous reviews.

## Methods

### Protocol and Guidance

We conducted a systematic review of randomized controlled trials (RCTs) according to the Preferred Reporting Items for Systematic Reviews and Meta-Analysis Protocol guidelines (18, 19) and the Cochrane Handbook for Systematic Reviews of Intervention (20). The protocol for this review has been registered with PROSPERO (CRD42020211070).

### Outcomes

The primary outcomes were as follows: (1) BPD (defined as oxygen dependency for 28 days or oxygen dependency at 36 weeks' PMA) in survivors (21); (2) moderate to severe BPD (defined as oxygen dependency and/or positive airway pressure requirements at 36 weeks' PMA) (21); (3) mortality (death before 1 month or at 36 weeks' PMA); (4) the composite incidences of mortality and oxygen requirement before 1 month or at 36 weeks' PMA; (5) effective vitamin A [defined as serum retinol concentration was statistically different between vitamin A and control groups at 28 days postnatal age (PNA)] supplementation for prevention of BPD and mortality.

The secondary outcomes were as follows: (1) length of hospital stay; (2) plasma retinol and vitamin A deficiency at 28 days PNA (define as serum retinol concentration <20 μg/dL) (22); (3) IVH of any grade or IVH of grade 3 or 4; (4) PVL; (5) IVH of grade 3–4 or PVL; (6) ROP of any grade or ROP requiring treatment; (7) NEC ≥stage 2; (8) sepsis; (9) adverse effects of vitamin A supplementation.

### Search Strategy

PubMed, EMBASE, Web of Science, and Cochrane Central Register of Controlled Trials were searched before 1 January, 2022. Keywords were established after an expert opinion was obtained, a literature review was completed, and controlled vocabulary (Medical Subject Headings and Excerpta Medica Tree) was used.

Search terms included “infant, very low birth weight”; “very low birth weight”; “birth weight, very low or very low birth weight infant or very low birthweight or VLBW”; “vitamin A”; “aquasol or retinol or All-Trans-Retinol or All Trans Retinol or Vitamin A1 or 11-cis-Retinol”; and “Controlled Clinical Trials or controlled clinical trial or randomized or placebo or randomly or trial”. The search strategy is presented in Supplementary 1.

### Study Selection

**Figure 1:**
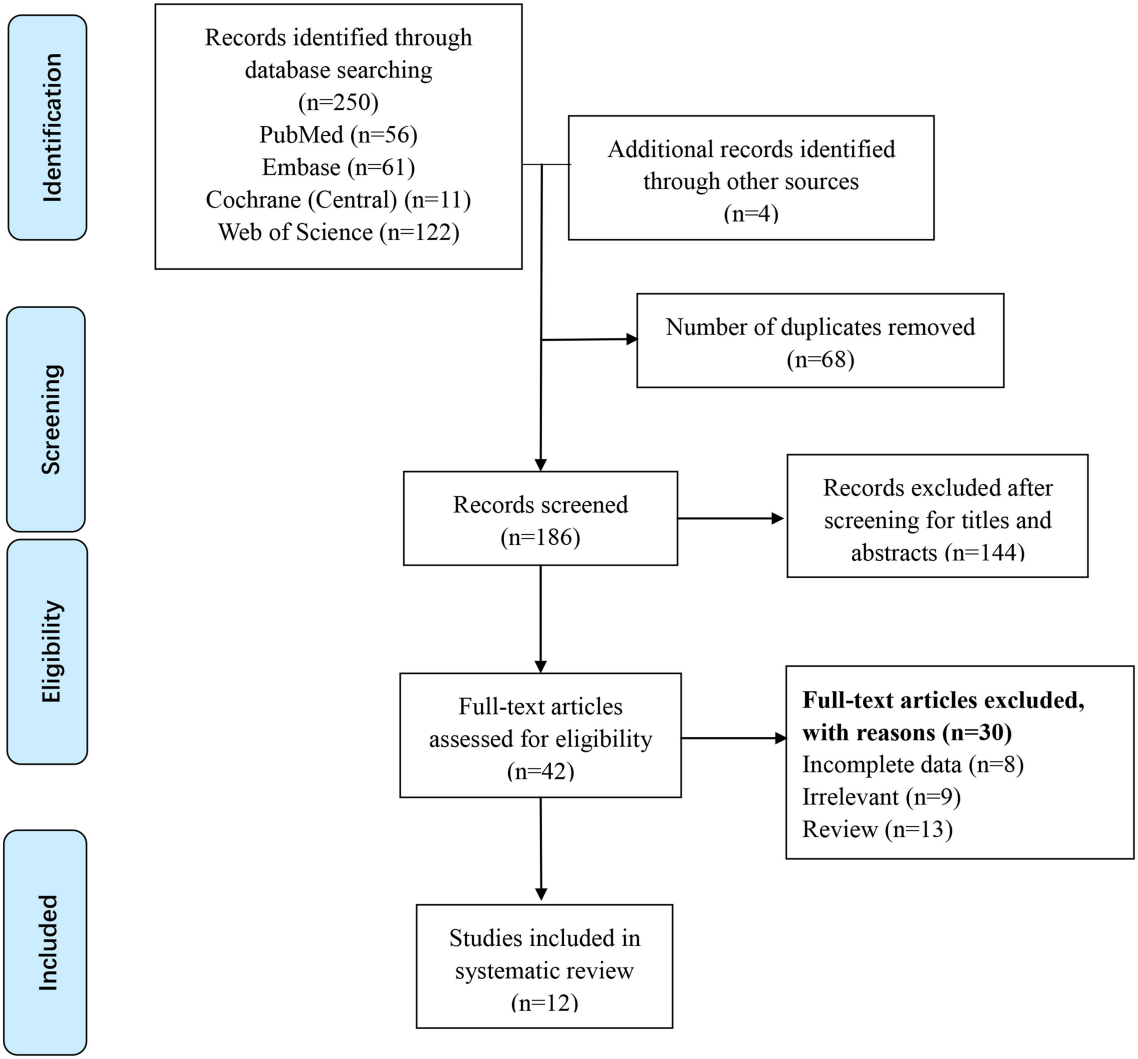
Flow diagram of search results and study selection.

After removing duplicates, two reviewers (YYX and HJH) independently selected articles for review by screening the titles and abstracts of all relevant studies identified in the search. When details of abstracts were ambiguous, the full text was also reviewed (Figure 1). Disagreement between reviewers regarding the selection of a study was resolved by consensus (JZ). The study inclusion criteria were as follows: (1) preterm infants with birth weight of <1,500 g and (2) RCTs comparing the effects of vitamin A vs. control in premature infants. The study exclusion criteria were as follows: (1) trials written in languages other than English and (2) trials with only abstracts and no obtainable original text or data.

### Data Extraction and Management

Data were independently extracted by two reviewers. Information extracted included the name of the first author, publication year, country of the participants, study design, sample size, gestational age and/or birth weight, treatment and control interventions, outcomes, and adjusted confounders.

### Risk of Bias Assessment

The two reviewers independently used the Cochrane Handbook for Systematic Reviews of Interventions to assess the risk of bias for all included studies (23). Disagreements were resolved through discussions between reviewers or by obtaining opinions from a third evaluator.

### Data Synthesis and Statistical Methods

Data were analyzed using Review Manager software (version 5.3). Statistical heterogeneity was evaluated using the *I*^2^ test. As all studies did not share a common true treatment effect and similar intervention characteristics (such as dose and modality) in included studies, the assumption of a common effect that might modify the treatment effect was not true (24–26). Thus, the fixed-effect model was not suitable and a random-effect model (Mantel–Haenszel method) was applied for all conditions. Dichotomous data were analyzed by determining RRs with 95% CIs, and continuous data were analyzed using mean differences (MDs) with 95% CIs. Statistical significance was set at *P*-value of <0.05. If the outcome was reported ≥10 trials, publication bias was assessed by visual measurement of the distribution and symmetry of funnel plot.

The subgroup analyses based on the methods of vitamin A supplementation. For results with high heterogeneity, sensitivity analysis was carried out to investigate the heterogeneity by excluding study with a high risk of bias.

We used GRADEpro software (http://gradepro.org) and Grade Handbook to assess quality of the evidence. The certainty of evidence was graded as high moderate, low, and very low (27).

### Ethical Approval

The data used in this manuscript were retrieved from public databases and previous studies; hence, ethics committee approval was not required. Further, there was no need for obtaining informed consent from patients.

## Results

### Search Results

We identified 254 potential studies: 56 from PubMed, 61 from EMBASE, 11 from CENTRAL, 122 from Web of Science, and 4 additional studies from associated references. Of these, 68 duplicate reports were removed, and 186 reports were screened. After careful screening, 12 studies were included in the final meta-analysis (12–15, 28–35). Key characteristics of the included studies are listed in Table 1. Almost all neonates included in this review were very preterm or extremely preterm infants, and the baseline vitamin A intake was the same between the two groups in the same study.

**Table 1:** Study characteristics.

**Reference**	**Country**	**Sample size (vit A /control)**	**Birth weight /g or gestational age**	**Interventions**			**Baseline vitamin A intake**	**Outcomes**
				**Vitamin A**	**Control**	**Administration time**		
Basu et al. (12)	India	98/98	<1,500	10,000 IU	Placebo solution	Orally on alternate days for 28 days	Parenteral nutrition and enteral feeding was are mentioned, but intakes of vitamin A was not stated	BPD; sepsis, NEC, IVH, PVL, ROP, serum retinol concentration, and vitamin A adverse effects
Giridhar et al. (15)	India	61/59	750 −1,250	5,000–10,000 IU	Normal saline and oral placebo	5,000 IU IM on alternate days till establishment of enteral feeds, followed by oral 10,000 IU daily for 28 days	Similar intakes of vitamin a from non-study sources but amounts not stated	BPD; plasma retinol, sepsis, IVH, PVL, and death
Kiatchoosakun et al. (35)	Thailand	40/40	<1,500 (24–32 weeks' gestation)	5,000 IU	Sham procedure	IM injection 3 times /week for four weeks	Enteral feeding was are mentioned, but intakes of vitamin A was not stated	BPD; serum vitamin A level, sepsis, NEC, ROP, and IVH
Mactier et al. (34)	UK	42/47	<1,501 (24-33 weeks' gestation)	10,000 IU	Mock injection	IM injection 3 times weekly till commencement of oral supplementation on day 14, or for a maximum of 12 doses	920 IU/kg/d vitamin A in parenteral nutrition and an supplement of 5,000 IU/d when fed orally	BPD; serum retinol concentration, IVH, and ROP
Pearson et al. (29)	USA	27/22	700–1,100	2,000 IU	Saline placebo	IM injection on alternate days for 14 doses.	1,200 to 1,500 IU/d vitamin A in parenteral nutrition and an supplement of 250 to 1,030 IU/dL when fed orally	BPD; length of hospital stay
Rakshasbhuvankar et al. (14)	Australia	94/94	<28 weeks' gestation	5,000 IU	Placebo solution	Orally daily until 34 weeks' PMA	966 IU/kg/d vitamin A in parenteral nutrition and an supplement of 1,820 IU/kg/d when fed orally	BPD; death, plasma retinol, ROP, sepsis, NEC, and vitamin A adverse effects
Ravishankar et al. (33)	USA	22/18	500–1,500 (24–30 weeks' gestation)	1,500–3,000 IU	Adhesive bandage	IM injection on days 1,3, and 7	466 IU/dL vitamin A in parenteral nutrition and an additional supplement of about 1,000 IU/d when fed orally	BPD; NEC, IVH, sepsis, and death
Shenai et al. (28)	USA	20/20	700–1,300	2,000 IU	Saline placebo	IM injection on alternate days for 14 doses	400 IU/dL vitamin A in parenteral nutrition and 240 to 550 IU/dL from milk plus 1,500 IU supplements when fed orally	BPD; length of hospital stay, IVH, and ROP
Sun et al. (13)	China	132/130	<28 weeks' gestation	1,500 IU	Placebo solution	Orally daily for 28 days or until discharge	230 IU/kg/d vitamin A in parenteral nutrition and an supplement of 2,568 or 1,764 IU/d when fed orally	BPD; serum retinol, ROP, sepsis, NEC, IVH, PVL, and vitamin A adverse effects
Tyson et al. (31)	USA	405/402	401–1,000	5,000 IU	Sham procedure	IM injection 3 times /week for 4 weeks	Similar intakes of vitamin A from non-study enteral and parenteral sources but amounts not stated	chronic lung disease; serum retinol, sepsis, IVH, PVL, and NEC
Wardle et al. (32)	UK	77/77	<1,000	5,000 IU	Placebo solution	Orally daily for 28 days	23 IU/kg/d vitamin A in parenteral nutrition and an supplement of 5,000 IU/kg/d when fed orally	BPD; serum retinol concentration, ROP, IVH, NEC, sepsis, and adverse effects
Werkman et al. (30)	USA	44/42	725–1,300 (24–31 weeks' gestation)	80,000 RE/L	Lipid emulsion	Intravenous lipids for 4 weeks	210 RE/d (birth weight <1,000 g, 1.5 mL/d) or 476 RE/d (> 1,000 g, 3.4 mL/d) vitamin A in parenteral nutrition and oral supplements when fed orally	BPD; plasma retinol

*BPD, bronchopulmonary dysplasia; PMA, postmenstrual age; IM, intramuscularly; IVH, intraventricular hemorrhage; PVL, periventricular leukomalacia; ROP, retinopathy of prematurity; NEC, necrotizing enterocolitis*.

### Assessment of Risk of Bias of Included Trials

**Figure 2:**
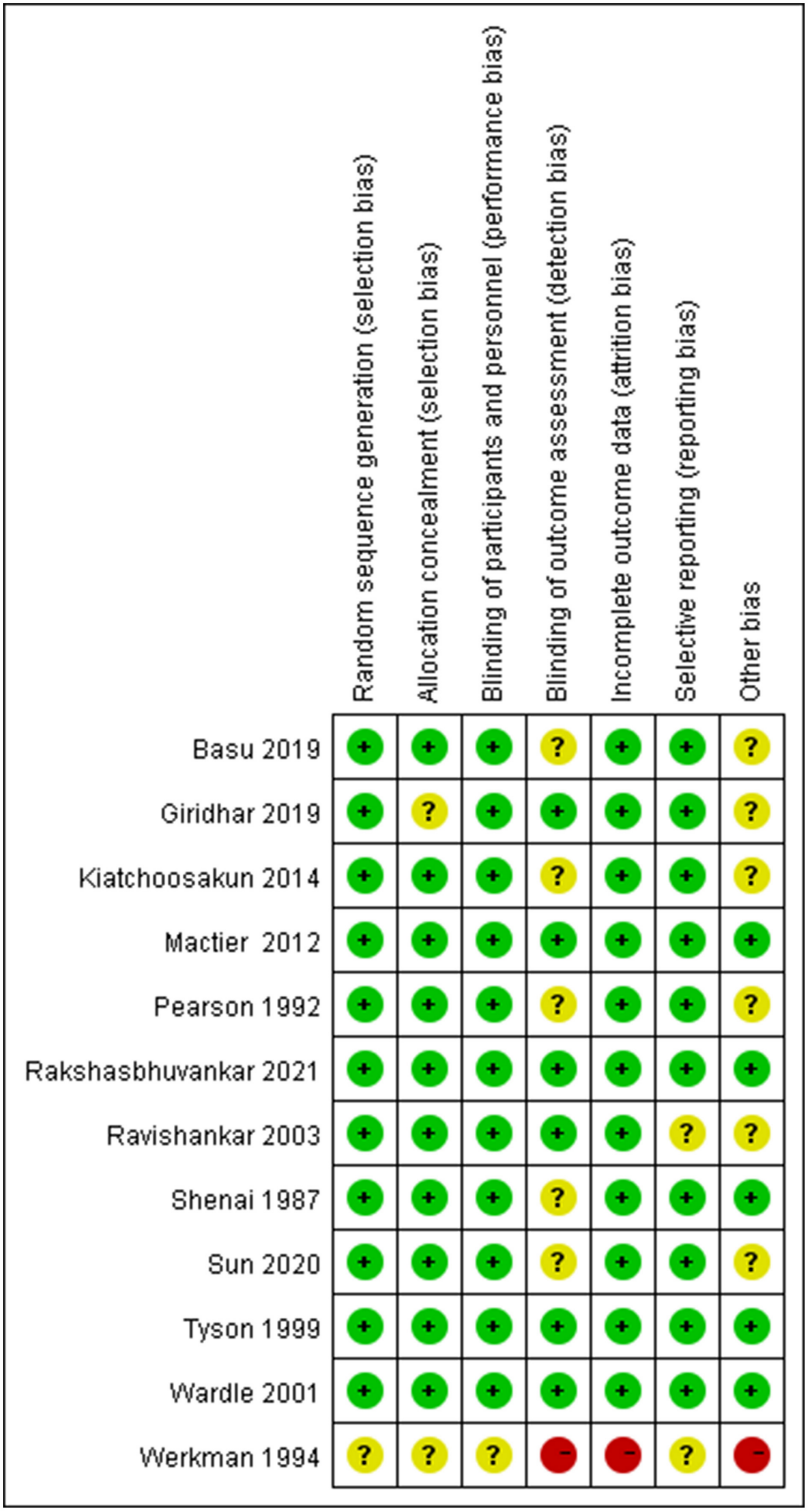
Risk of bias summary.

The summary of the risk of bias assessment for each study is shown in Figure 2 and Supplementary 2 show the support quotes for the judgement of risk of bias.

### Interventions

For included studies, vitamin A supplementation was compared with either the provision of a placebo or no treatment. Vitamin A was administered intramuscularly (IM) (6 studies), orally (4 studies), intravenously (1 study) or IM followed with subsequent oral administration (1 study). In four studies, the vitamin A dosage provided 5,000 IU IM every other day for 12 doses, or 5,000 IU/day orally for 28 days or until 34 week's PMA (14, 31, 32, 35). Alternatively, 10,000 IU of oral or IM vitamin A supplementation on alternate days was administered to patients in the studies by Basu et al. (12) and Atkins et al. (27). In the study by Giridhar et al., 5,000 IU of vitamin A was administered by IM on alternate days until adequate enteral feeds have been established. Then, 10,000 IU of oral vitamin A was provided daily for 28 days. Alternately, 1,500–3,000 IU of vitamin A supplementation IM or orally was provided in four studies (14, 21, 22, 26), whereas 80,000 RE/L (equivalent to 1300–3300 IU/kg/d) of vitamin A administered via intravenous lipids was provided in Higgins et al. (23).

### Outcomes

In this review, 12 RCTs and a total of 2,111 premature neonates were eligible for analysis. Among the 2,111 neonates, 1,062 were in the intervention group and 1,049 were in the control group. All outcomes are shown in Table 2. None of the outcome was reported ≥10 trials, and publication bias was not assessed.

**Table 2:** The main results of vitamin A supplementation and its effects.

**Outcomes**	**Studies**	**Pooled RR/** **MD (95% CI)**	***I*** **^2^ /%**	***p***
Oxygen dependency			
For 28 days At 36 weeks' PMA	7 9	0.89 (0.76–1.06) 0.84 (0.69–1.03)	41 46	0.19 0.09
Moderate to severe BPD	9	0.86 (0.71–1.03)	40	0.11
Mortality			
Before 1month At 36 weeks' PMA	6 6	0.86 (0.62–1.19) 0.95 (0.75–1.21)	17 0	0.36 0.67
Composite incidence			
Before 1month At 36 weeks' PMA	6 6	0.89 (0.77–1.04) 0.93 (0.81–1.08)	58 43	0.13 0.34
Effective vitamin A supplementation			
BPD before 1month BPD at 36 weeks' PMA Death before 1month Death at 36 weeks' PMA	3 7 3 4	0.69 (0.32–1.47) 0.79 (0.60–1.04) 0.90 (0.50–1.64) 0.99 (0.74–1.33)	57 50 45 0	0.33 0.09 0.74 0.94
Length of hospital stay	6	−12.67 (-23.55 to −1.79)	97	0.02
Plasma retinol at 28 days Vitamin A deficency	5 3	24.74 (6.62–42.87) 0.08 (0.02–0.38)	98 67	0.007 0.001
IVH			
Any grade Grade 3 or 4	3 5	0.94 (0.80–1.09) 0.89 (0.68–1.15)	0 0	0.40 0.36
PVL			
IVH (grade 3or 4) or PVL	4 3	0.68 (0.47–0.97) 0.85 (0.69–1.05)	0 0	0.03 0.13
ROP			
Any grade Requiring treatment	4 5	0.61 (0.48–0.76) 0.83 (0.43–1.61)	0 16	<0.0001 0.59
NEC	7	0.91 (0.68–1.22)	0	0.52
Sepsis	6	0.91 (0.79–1.05)	0	0.18

*RR, risk ratio; MD, mean difference; CI, confidence intervals; BPD, bronchopulmonary dysplasia; PMA, postmenstrual age; IVH, Intraventricular hemorrhage; PVL, Periventricular leukomalacia; ROP, Retinopathy of prematurity; NEC, Necrotizing enterocolitis*.

### Primary Outcomes

#### Vitamin A Supplementation and BPD

##### Oxygen Dependency for 28 Days in Survivors

**Figure 3:**
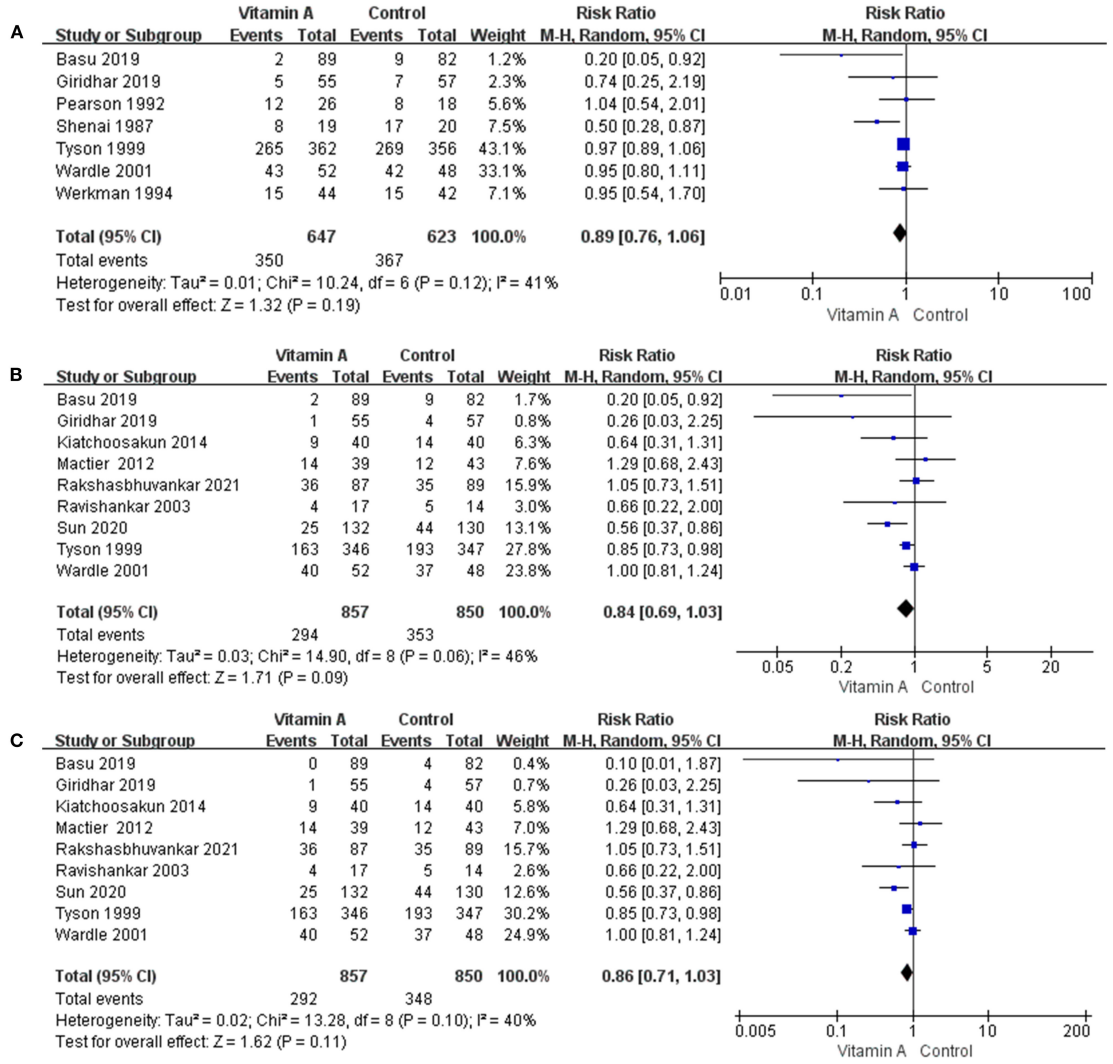
The forest plot for the incidences of oxygen dependency among survivors. **(A)** The forest plot for the incidence of oxygen dependency for 28 days among survivors. **(B)** The forest plot for the incidence of oxygen dependency at 36 weeks' PMA among survivors. **(C)** The forest plot for the incidence of moderate to severe BPD among survivors.

Seven eligible studies reported oxygen dependency for 28 days. The requirement of oxygen for 28 days among survivors was 54.10% (*n* = 350/647) for VLBW infants receiving vitamin A compared to 58.91% (*n* = 367/623) for those in the control group. However, the meta-analysis revealed no reduction in the incidence of oxygen use for 28 days associated with vitamin A supplementation (RR: 0.89; 95% CI: 0.76–1.06; *p* = 0.19; *I*^2^ = 41%; Figure 3A).

##### Oxygen Dependency at 36 Weeks' PMA in Survivors

Nine eligible studies provided data on oxygen dependency at 36 weeks' PMA. The oxygen support requirement at 36 weeks' PMA among survivors was 36.53% (*n* = 293/802) in the vitamin A group. In the control group, oxygen was required at 36 weeks' PMA in 44.01% (*n* = 349/793) of VLBW infants. However, there was no significant reduction in the incidence of BPD with oxygen dependency at 36 weeks' PMA (RR: 0.84; 95% CI: 0.69–1.03; *p* = 0.09; *I*^2^ = 46%; Figure 3B).

##### Moderate to Severe BPD

Nine eligible studies provided data on moderate to severe BPD. The incidences of moderate to severe BPD was 34.07% (*n* = 292/857) in the vitamin A group and 40.94% (*n* = 348/850) in the control group. However, the difference between the two groups was not statistically significant (RR: 0.86; 95% CI: 0.71–1.03; *p* = 0.11; *I*^2^ = 40%; Figure 3C).

#### Vitamin A Supplementation and Mortality

##### Mortality (Death Before 1 Month)

**Figure 4:**
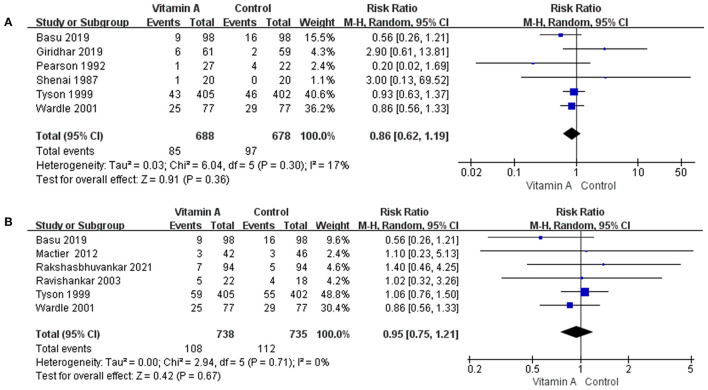
The forest plot for the incidences of mortality. **(A)** The forest plot for the incidence of death before 1 month. **(B)** The forest plot for the incidence of death at 36 weeks' PMA.

Six eligible studies reported data on the mortality before 1 month. Mortality before 1 month was 12.35% (*n* = 85 /688) in the vitamin A group and 14.31% (*n* = 97/678) in the control group. The difference between the two groups was not statistically significant (RR: 0.86; 95% CI: 0.62–1.19; *p* = 0.36; *I*^2^ = 17%; Figure 4A).

##### Mortality (Death at 36 Weeks' PMA)

Six eligible studies reported data on the mortality at 36 weeks' PMA. In the group that received vitamin A, mortality at 36 weeks' PMA was 14.63% (*n* = 108/738), while mortality at 36 weeks' PMA was 15.24% (*n* = 112/735) for the control group. However, the difference was not statistically significant (RR: 0.95; 95% CI: 0.75–1.21; *p* = 0.67; *I*^2^ = 0%; Figure 4B).

#### Composite Incidences of Mortality and Oxygen Requirement Before 1 Month

**Figure 5:**
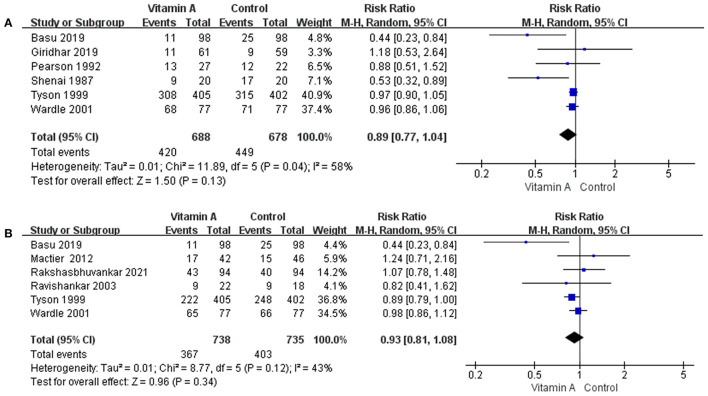
The forest plot for the composite incidences of mortality and oxygen requirement. **(A)** The forest plot for the composite incidences of mortality and oxygen requirement before 1 month. **(B)** The forest plot for the composite incidences of mortality and oxygen requirement at 36 weeks' PMA.

The composite incidences of mortality and oxygen requirement before 1 month was 61.05% (*n* = 420/688) in the vitamin A group and 66.22% (*n* = 449/678) in the control group. However, the difference between the two groups was not statistically significant (RR: 0.89; 95% CI: 0.77–1.04; *p* = 0.13; *I*^2^ = 58%; Figure 5A).

#### Composite Incidences of Mortality and Oxygen Requirement at 36 Weeks' PMA

The composite incidences of mortality and oxygen requirement at 36 weeks' PMA was 49.73% (*n* = 367/738) in the vitamin A group and 54.83% (*n* = 403/735) in the control group. However, the difference between the two groups was not statistically significant (RR: 0.93; 95% CI: 0.81–1.08; *p* = 0.34; *I*^2^ = 43%; Figure 5B).

#### Effective Vitamin A Supplementation for Prevention of BPD and Mortality

Nine eligible studies reported plasma retinol at 28 days PNA, and seven studies reported statistical differences in plasma vitamin A levels between the two groups (12–15, 31, 34, 35). However, effective vitamin A supplementation did not reduce the incidence of BPD or death in VLBW infants (Supplementary Figure 1).

### Secondary Outcomes

Vitamin A supplementation may reduce the duration of hospitalization of VLBW infants, meanwhile improve the plasma retinol and reduce the incidence of VAD in preterm infants. However, the statistical heterogeneity was considerable, and sensitivity and subgroup analyses could not explain inconsistency in results. We conducted in-depth analysis of this result in our discussion.

### Length of Hospital Stay

Eight eligible studies provided data on the length of hospital stay. The length of hospitalization data in the trials by Werkman et al. (30) and Tyson et al. (31) could not be synthesized; hence, they were excluded from the analysis. The length of hospital stay was significantly shorter in the vitamin A group than in the control group (mean difference [MD], −12.67; 95% CI −23.55 to −1.79; *p* = 0.02; *I*^2^ = 97%; Supplementary Figure 2).

### Plasma Retinol

Nine eligible studies reported plasma retinol at 28 days PNA. The plasma retinol data in the trials of Higgins et al. (23), Nikolakopoulou et al. (24), and Atkins et al. (27) could not be synthesized; and the data reported by Borenstein et al. (25) could not be extracted effectively; thus, we excluded these results from the analysis. Plasma retinol levels were significantly higher in the vitamin A group than in the control group (MD, 24.74; 95% CI 6.62–42.87; *p* = 0.007; *I*^2^ = 98%; Supplementary Figure 3A).

Three eligible studies reported vitamin A deficiency at 28 days PNA. The incidence of VAD was significantly different between the vitamin A and control groups (RR: 0.08; 95% CI: 0.02–0.38; *p* = 0.001; *I*^2^ = 67%; Supplementary Figure 3B).

### Intraventricular Hemorrhage (IVH)

Three eligible studies provided data on the incidence of IVH of any grade, whereas five eligible studies provided the incidence of IVH of grade 3 or 4. There was no significant difference observed between the vitamin A and control groups in the incidence of IVH of any grade (RR: 0.94; 95% CI: 0.80–1.09; *p* = 0.40; *I*^2^ = 0%; Supplementary Figure 4A) or IVH of grade 3 or 4 (RR: 0.89; 95% CI: 0.68–1.15; *p* = 0.36; *I*^2^ = 0%; Supplementary Figure 4B).

### Periventricular Leukomalacia (PVL)

Four eligible studies reported incidence of PVL. The incidence of PVL was significantly different between the vitamin A and control groups (RR: 0.68; 95% CI: 0.47–0.97; *p* = 0.03; *I*^2^ = 0%; Supplementary Figure 5A). Three eligible studies reported on the incidence of IVH grade of 3–4 or PVL. There was no significant difference between the incidence of IVH of grade 3–4 or PVL when the vitamin A group and the control group were compared (RR: 0.85; 95% CI: 0.69–1.05; *p* = 0.13; *I*^2^ = 0%; Supplementary Figure 5B).

### Retinopathy of Prematurity (ROP)

Four eligible studies reported the incidence of ROP of any grade and five eligible studies reported the incidence of ROP requiring treatment. The incidence of ROP of any grade was significantly different between the vitamin A and control groups (RR: 0.61; 95% CI: 0.48–0.76; *p* <0.0001; *I*^2^ = 0%; Supplementary Figure 6A). However, no significant difference between the incidence of ROP requiring treatment was observed (RR: 0.83; 95% CI: 0.43–1.61; *p* = 0.59; *I*^2^ = 16%; Supplementary Figure 6B).

### Necrotizing Enterocolitis (NEC)

Seven eligible studies reported data on the incidence of NEC. When the vitamin A and control groups were compared, no significant difference in the incidence of NEC was observed (RR: 0.91; 95% CI: 0.68–1.22; *p* = 0.52; *I*^2^ = 0%; Supplementary Figure 7A).

### Sepsis

Six eligible studies reported data on the incidence of sepsis. There was no significant difference between the incidence of sepsis when the vitamin A and control groups were compared (RR: 0.91; 95% CI: 0.79–1.05; *p* = 0.18; *I*^2^ = 0%; Supplementary Figure 7B).

### Adverse Effects of Vitamin A Supplementation

Eight eligible studies reported the safety of vitamin A supplementation. Both Basu et al. (12) and Wardle et al. (32) reported possible adverse events, such as seizures or vomiting. However, the incidence of these effects in both groups was similar. The incidence of vomiting was 2.29% (*n* = 4/175) in the vitamin A group and 2.86% (*n* = 5/175) in the control group. The incidence of seizure was 19.48% (*n* = 15/77) in the vitamin A group and 25.97% (*n* = 20/77) in the control group. Tyson et al. (31) reported an increase in the fontanelle tension in the control group compared with that in the vitamin A group (18% vs. 15%, *p* = 0.26). They also reported that potential vitamin A toxicity of 1.0% in the vitamin A group and 0.8% in the control group was unexplained by other factors (e.g., post-haemorrhagic hydrocephalus causing a full fontanelle). Giridhar et al. (15) also reported that two infants in the vitamin A group had unusual high plasma retinol levels with no clinical signs of toxicity, and the other eligible studies reported no signs of potential vitamin A toxicity.

### Subgroup Analyses

**Figure 6:**
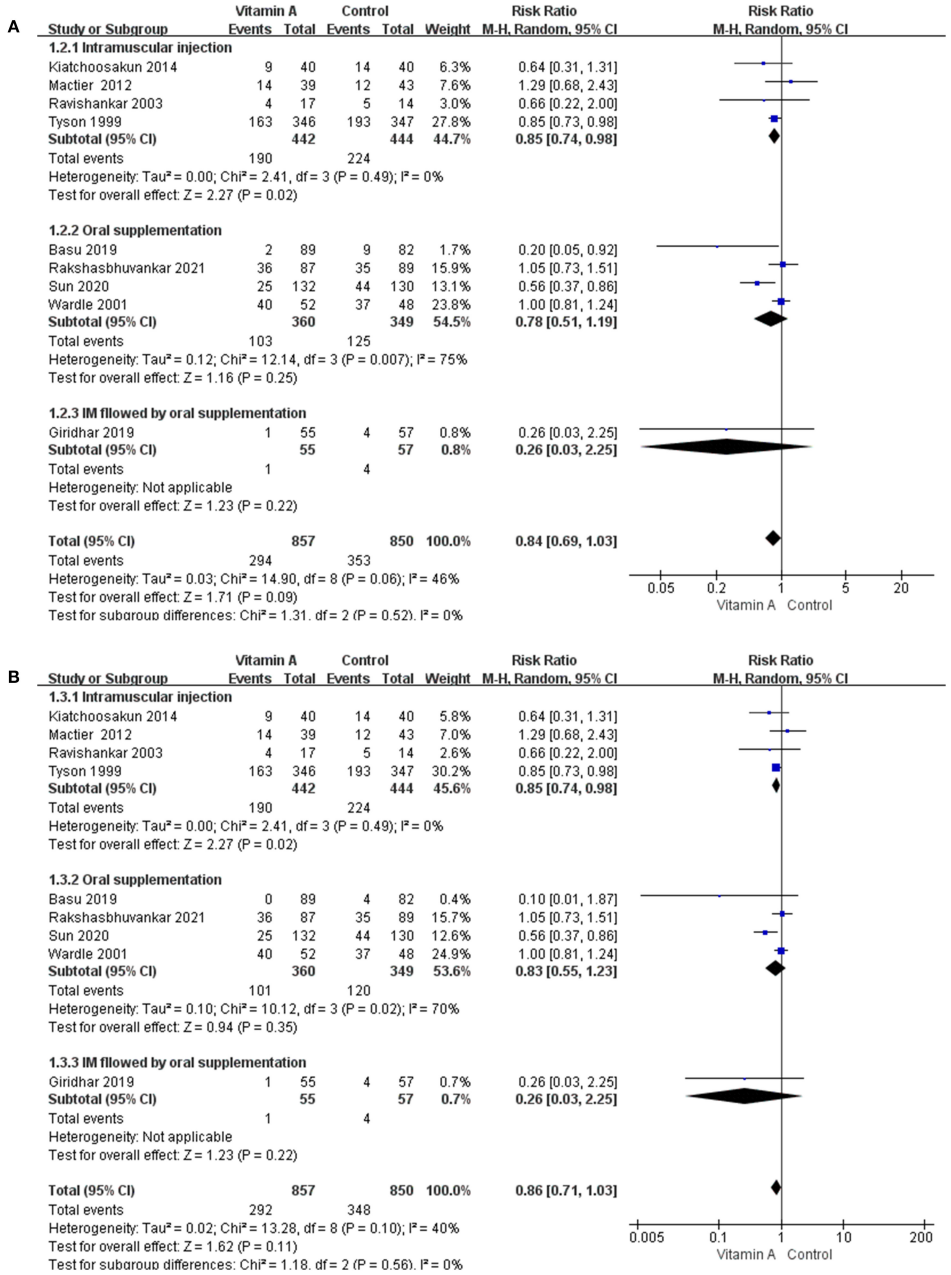
The subgroup analyses based on the methods of vitamin A supplementation. **(A)** The forest plot for the incidences of BPD at 36 weeks' PMA. **(B)** The forest plot for the incidences of moderate to severe BPD.

The subgroup analyses based on the methods of vitamin A supplementation did not alter the pooled results for any outcomes. However, there were subgroup differences in some important outcomes. The test for subgroup differences suggested a subgroup difference for the incidence of BPD at 36 weeks' PMA (Figure 6A) and the incidence of moderate to severe BPD (Figure 6B). The subgroup analysis showed that vitamin A supplementation reduced the incidence of BPD at 36weeks' PMA (RR and moderate to severe BPD only in subgroup with intramuscular injection (0.85, 95% CI: 0.74–0.98; *p* = 0.02; *I*^2^ = 0%).

### Sensitivity Analyses

Sensitivity analyses conducted after excluding studies with a possible high risk of bias did not change any of the outcomes.

### Certainty of Evidence

The certainty of evidence for the outcomes ranged from moderate to very low. All outcomes are shown in the summary of findings table (Table 3).

**Table 3:** Summary of findings.

**Vitamin A supplementation compared with placebo for morbidity and mortality in VLBW infants**						
**Patient or population: VLBW infants. Intervention: Vitamin A. Comparison: placebo**						
**Outcomes**	Anticipated absolute effects* (95% CI)		**Relative effect (95% CI)**	**No. of participants (studies)**	**Certainty of the evidence (GRADE)**	**Comments**
	**Risk with placebo**	**Risk with Vitamin A**				
BPD at 36 weeks' PMA	440 per 1,000	374 per 1,000 (308 to 458)	RR 0.85 (0.70 to 1.04)	1,595 (8 RCTs)	⊕⊕○○ Low^a, b^	
Mortality or BPD at 36 weeks' PMA	152 per 1,000	145 per 1,000 (114 to 184)	RR 0.95 (0.75 to 1.21)	1,473 (6 RCTs)	⊕⊕⊕○ Moderate^b^	
Moderate to severe BPD	409 per 1,000	352 per 1,000 (291 to 422)	RR 0.86 (0.71 to 1.03)	1,707 (9 RCTs)	⊕⊕○○ Low^a, b^	
Length of hospital stay	-	MD 12.67 fewer (23.55 fewer to 1.79 fewer)	-	739 (6 RCTs)	⊕○○○ Very low^c, d^	
Vitamin A deficiency	486 per 1,000	39 per 1,000 (10 to 185)	RR 0.08 (0.02 to 0.38)	358 (3 RCTs)	⊕⊕○○ Low^d, e^	
PVL	106 per 1,000	72 per 1,000 (50 to 103)	RR 0.68 (0.47 to 0.97)	1,224 (4 RCTs)	⊕⊕○○ Low^d, f^	
ROP of any grade	494 per 1,000	301 per 1,000 (237 to 375)	RR 0.61 (0.48 to 0.76)	463 (4 RCTs)	⊕⊕⊕○ Moderate^d^	

* *The risk in the intervention group (and its 95% confidence interval) is based on the assumed risk in the comparison group and the relative effect of the intervention (and its 95% CI)*.

*CI, confidence interval; MD, mean difference; RR, risk ratio; BPD, bronchopulmonary dysplasia; PMA, postmenstrual age; PVL, Periventricular leukomalacia; ROP, Retinopathy of prematurity*.

*GRADE Working Group grades of evidence*.

*High certainty, we are very confident that the true effect lies close to that of the estimate of the effect*.

*Moderate certainty: we are moderately confident in the effect estimate: the true effect is likely to be close to the estimate of the effect, but there is a possibility that it is substantially different*.

*Low certainty: our confidence in the effect estimate is limited: the true effect may be substantially different from the estimate of the effect*.

*Very low certainty: we have very little confidence in the effect estimate: the true effect is likely to be substantially different from the estimate of effect*.

a *The subgroup difference was inconsistent*.

b *95% CI is wide and fails to exclude important benefit or harm*.

c *Considerable heterogeneity: minimal overlaps of CIs*.

d *The optimal information size criterion was not met*.

e *Substantial heterogeneity may be considered*.

f *Wide variance of point estimates across studies*.

## Discussion

This review includes 12 RCTs to evaluate the effects of vitamin A supplementation on short-term morbidity and mortality in VLBW infants. The study revealed that vitamin A supplementation in premature infants reduces the incidence of VAD (low certainty of evidence), PVL (low certainty of evidence), and ROP of any grade (moderate certainty of evidence). The difference in length of hospital stay was significant between two groups. However, no significant differences were observed in the incidence of BPD or moderate to severe BPD, death before 1 month or at 36 weeks' PMA, and other accompanying complications.

Previous meta-analyses showed that vitamin A supplementation in preterm infants could reduce the incidence of BPD. A network meta-analysis published in 2017 (36) evaluated the effects of seven single treatments (including beclomethasone, budesonide, caffeine, dexamethasone, fluticasone, hydrocortisone, and vitamin A) and one combined treatment (budesonide+caffeine) on mortality and morbidity among preterm infants. The results revealed that vitamin A had the highest impact on the duration of ventilation, oxygen supplementation, and hospitalization. Four recent meta-analyses showed that vitamin A supplementation could reduce oxygen dependency at 36 weeks' PMA among VLBW infants (11) and ELBW infants (17, 37), reduce the incidence of BPD in premature infants (38), and shorten the length of hospital stay (17). The differences in the results are attributed to the following: first, we included 12 RCTs, including four very recent studies, more than the previous meta-analyses. It is worth noting that most of the RCTs included in the previous meta-analyses were published before the year 2014, and the major trials by Tyson et al. (31) and Wardle et al. (32) were conducted two decades ago. In the study by Tyson et al. and Wardle et al., the incidence of moderate to severe BPD was relatively high in both treatment and control groups (Tyson et al.: 47% vs. 55%, Wardle et al.: 76.9% vs. 77.1%). In recent years, the improvement in clinical management and the wide use of new lung protective strategies among preterm infants including non-invasive ventilation strategies, volume targeted ventilation, and early extubation; the use of less invasive surfactant administration technique; and early commencement of caffeine citrate have significantly reduced the severity of BPD (39), with the incidence of moderate to severe BPD in most recent studies being below 40%. Compared with studies conducted over 20 years ago, baseline vitamin A intake was higher in more recent studies due to the current dietary guidelines for preterm infants (40). General improved care of VLBW infants seems to have enhanced the effect of vitamin A on the prevention of BPD. Another reason for the difference in our findings is that more studies of oral vitamin A supplementation were included in our study. The subgroup analysis showed that vitamin A supplementation by intramuscular injection reduced the incidence of oxygen dependence at 36 weeks' PMA (RR 0.85, 95% CI: 0.74–0.98), but the oral counterparts did not (RR 0.78, 95% CI: 0.51–1.19). The *I*^2^ test result in the oral subgroup was more than 70%, which indicated that heterogeneity was greater for this group. This considerable heterogeneity may be due to the variations in the doses (ranging from 1,500 to 10,000IU), drug formulation (water-soluble form or fat-soluble form), and retinol concentration among the studies. The heterogeneity and small sample sizes of studies utilizing the oral administration route of vitamin A supplementation may have led to an inappropriate estimation of its true effect. More original studies are required to evaluate whether vitamin A has a protective effect in premature infants. Currently, one large-scale ongoing clinical trial in Germany is evaluating the safety and effectiveness of oral vitamin A supplementation in preventing BPD in VLBW infants (41). This trial will administer 5,000 IU of vitamin A orally per day to a total of 1,100 neonates that are enrolled in the study (42, 43). More relevant studies in the future will make it easier to discuss and determine the effectiveness of vitamin A supplementation.

ROP is a vasoproliferative vitreoretinal disorder arising from incomplete or immature retinal vascularisation in preterm infants. It is also one of the major causes of blindness in premature infants. As an antioxidant, vitamin A can regulate the expression of vascular endothelial growth factor (VEGF) (44). Agrawal et al. reported that VAD in the umbilical cord blood of preterm infants was one of the independent risk factors for ROP (8). In addition, animal studies have shown that retinoic acid supplementation could regulate expression of VEGF and prevent hyperoxia-induced retinal angiogenesis (44). Mactier et al. (34) also demonstrated that early IM vitamin A supplementation for infants at risk of ROP improved retinal function at 36 weeks' PMA. These studies indicate that vitamin A may have a potentially protective effect on ROP. Our study found that early vitamin A supplementation in preterm infants can prevent ROP at any grade, which differs from the findings from previous meta-analyses aimed at evaluating the preventive effect of vitamin A on BPD. Most studies included in these meta-analyses had not used ROP as the primary outcome measure, which may have led to underestimation of the effect of vitamin A on the prevention of ROP. The largest RCT on the use of vitamin A supplementation to prevent ROP thus far was published in 2020 (13). This study examined 262 ELBW infants and found a reduction in the incidence of both mild and severe ROP in the treatment group. The results of our meta-analysis are similar to those of the two previous meta-analysis that estimated the effects of vitamin A in the prevention of ROP. These meta-analyses including both randomized and observational studies showed that vitamin A supplementation could reduce the risk of ROP at any stage (16, 45).

One notable finding of our study was that the incidence of PVL in the vitamin A group was lower than that in the control group. PVL is a common form of brain injury in preterm infants. Its primary pathogenesis includes maturation-dependent vulnerability of oligodendrocyte precursor cells. Developing oligodendrocytes are very sensitive to oxidative stress and, as an antioxidant, vitamin A plays an important role in promoting the development of brain gangliosides and myelination (46, 47). Postnatal VAD can lead to selective memory impairment in mice (48), learning and spatial memory deficits in rats (49), and an increased risk of delayed neurodevelopment at 1 year and 2 years of corrected age among VLBW infants (9). Zhang et al. revealed that the concentration of retinol in cord blood was positively correlated with postnatal social and motor function at 2 years of age (50). Retinoic acid, a metabolic product of vitamin A, was able to induce neural stem cells to differentiate toward the oligodendrocyte lineage in both *in vivo* and *in vitro* rat models of focal demyelination (44, 45). In a small RCT, Strømmen et al. found that enhanced postnatal nutrient supply (increased amounts of energy, protein, fat, essential fatty acids and 1,500 μg/kg/day of vitamin A until discharge) in VLBW infants was beneficial to brain white matter maturation compared with a standard nutritional supply (51). A few previous meta-analyses evaluated the effects of vitamin A supplementation on PVL. A recent Cochrane systematic review only included one article and found no benefit, whereas our meta-analysis included four studies and found that vitamin A supplementation reduced the incidence of PVL. However, the sample size of each study was small, and more clinical trials are required to confirm the neuroprotective effect of vitamin A among VLBW infants.

The optimal vitamin A dose and retinol concentration for VLBW infants is not clear (9). The World Health Organization defines VAD as serum retinol concentration of <20 μg/dl and severe VAD as serum retinol concentration of <10 μg/dl (22). VAD is a common condition found in most VLBW infants (52). Tyson et al. (31) suggested that a vitamin A dose of 5,000 IU administered IM three times per week for 4 weeks could effectively increase the serum retinol levels in the treatment group 28 days' PNA, decrease the proportion of VAD, and reduce the incidence of BPD. Shenai (52) recommended that supplemental vitamin A should be administered by IM injection at 2000IU/kg/dose on alternate days until full enteral feeding is established, followed by a dose of 4000 IU/kg/day via orogastric administration. Landman et al. (53) also indicated that VAD could be corrected by enteral supplementation at a dose of 5,000 IU/day in most preterm infants who can tolerate oral feeding. However, the study by Sun et al. (13) administered a lower dose of oral vitamin A to ELBW infants at 1500 IU/day, which also resulted in reduction of ROP and BPD. The study conducted by Wardle et al., who enterally administered vitamin A at a dose of 5000 IU/day for 28 days, found no difference in the plasma retinol concentrations between the treatment and control groups at 28 days' PNA. Rakshasbhuvankar et al. (14) explains that these low retinol levels may be due to the poor enteral absorption of the fat-soluble form of vitamin A and that the water-soluble form may produce a higher retinol level. However, Sun et al. administered a lower dose of oral fat-soluble vitamin A to ELBW infants but was able to reach higher plasma retinol levels in their treatment group. We cannot explain these differences in retinol level and how they may or may not correlate to the different doses and dosage forms used across the studies in our analysis. To clarify the optimal dose and effect of vitamin A among VLBW infants, it may be necessary to conduct a homogenized international multi-center research.

Our study shows that the current vitamin A supplementation strategy is safe, even at relatively high doses. A retrospective cohort study conducted by Uberos et al. (54) indicated that intramuscular injections of vitamin A were associated with an increased risk of sepsis in patients weighing >1 kg. However, there was no significant difference in the incidence of sepsis between the vitamin A and control groups in our study. Thus, the safety of vitamin A supplementation for the prevention of BPD in VLBW infants requires further exploration.

This meta-analysis has some limitations. First, vitamin A was administered IM, orally, or IM followed by oral administration, and the dosages of vitamin A varied greatly among the eligible studies. The use of different administration methods and dosages of vitamin A supplementation may affect pharmacokinetics, and therefore may have decreased the reliability of our results. Second, the number of studies included in this analysis was small, and the trial that included the largest number of people was also the oldest study, which was conducted in 1999 (31). The changing definitions of BPD and the progress of medical technology add to limitations of the applicability of early studies, which reduce the number of appropriate studies available for assessment. We will update our study as a new large-scale clinical trial in Germany will be published in the future. Lastly, we restricted the language to English when we selected articles, which may have caused overlook of valuable articles published in other languages.

## Conclusion

This meta-analysis found no convincing evidence that vitamin A supplementation prevented BPD in VLBW infants. However, vitamin A supplementation was found to reduce the incidence of ROP of any grade as well as the length of hospital stay, and may have had an effect on PVL prevention. More large scale and adequately powered randomized studies are needed before recommending routine use of high dose of Vitamin A in very preterm infants to prevent BPD.

## Data Availability Statement

The original contributions presented in the study are included in the article/Supplementary Material, further inquiries can be directed to the corresponding author/s.

## Author Contributions

YY, JZ, and JH were responsible for literature search and retrieving data. XY, XX, and JS were responsible for design and concept of the manuscript. YY, JL, JS, and DM were responsible for the analysis and writing of the manuscript. All authors read and approved the final manuscript.

## Funding

This work was supported by the grants from the Science and Technology Bureau of Sichuan Province (2020YFS0041) and the Clinical research funding of West China Second University Hospital, Sichuan University (KL075).

## Conflict of Interest

The authors declare that the research was conducted in the absence of any commercial or financial relationships that could be construed as a potential conflict of interest.

## Publisher's Note

All claims expressed in this article are solely those of the authors and do not necessarily represent those of their affiliated organizations, or those of the publisher, the editors and the reviewers. Any product that may be evaluated in this article, or claim that may be made by its manufacturer, is not guaranteed or endorsed by the publisher.

## Supplementary Material

Supplementary Figure 1 Effective vitamin A supplementation for prevention of BPD and mortality. **(A)** The forest plot for the incidence of oxygen dependency for 28 days among survivors. **(B)** The forest plot for the incidence of oxygen dependency at 36 weeks' PMA among survivors. **(C)** The forest plot for the incidence of death before 1 month. **(D)** The forest plot for the incidence of death at 36 weeks' PMA.

Supplementary Figure 2 The forest plot for the length of hospital stay.

Supplementary Figure 3 **(A)** The forest plot for the plasma retinol. **(B)** The forest plot for the incidences of vitamin A deficiency.

Supplementary Figure 4 The forest plot for the incidences of IVH. **(A)** The forest plot for the incidence of IVH of any grade. **(B)** The forest plot for the incidence of IVH of grade 3 or 4.

Supplementary Figure 5 The forest plot for the incidences of PVL. **(A)** The forest plot for the incidence of PVL. **(B)** The forest plot for the incidence of IVH of grade 3 or 4 or PVL.

Supplementary Figure 6 The forest plot for the incidences of ROP. **(A)** The forest plot for the incidence of ROP of any grade. **(B)** The forest plot for the incidence of ROP requiring treatment.

Supplementary Figure 7 **(A)** The forest plot for the incidence of NEC. **(B)** The forest plot for the incidence of sepsis.

Supplementary 1 Search strategies.

Supplementary 2 Risk of bias assessment.

## Abbreviations

VLBW: very-low-birth-weight
ELBW: extremely-low-birth-weight
GRADE, Grading of Recommendations, Assessment: Development and Evaluation
BPD: bronchopulmonary dysplasia
RR: risk ratio
MD: mean difference
CI: confidence intervals
VAD: vitamin A deficiency
PMA: postmenstrual age
PNA: postnatal age
RCTs: randomized controlled trials
IM: intramuscularly
IVH: intraventricular hemorrhage
PVL: periventricular leukomalacia
ROP: retinopathy of prematurity
NEC: necrotizing enterocolitis
VEGF: vascular endothelial growth factor.
