# Supplementary figures and images for: Early Vitamin A Supplementation for Prevention of Short-Term Morbidity and Mortality in Very-Low-Birth-Weight Infants: A Systematic Review and Meta-Analysis
Source: Front Pediatr. 2022 Apr 7;10:788409. doi: 10.3389/fped.2022.788409 (PMC9021759; doi:10.3389/fped.2022.788409)

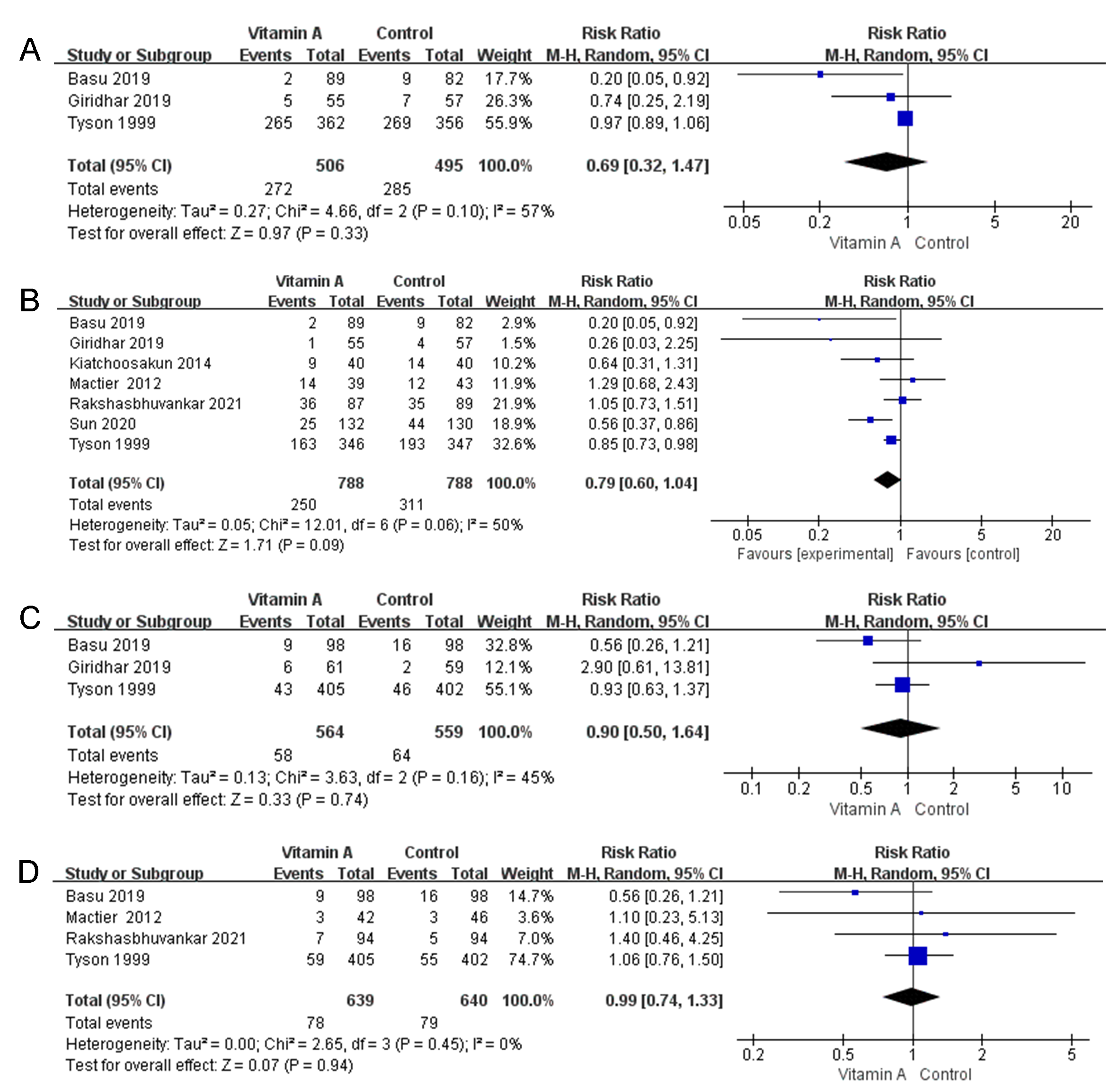

Supplement: Supplementary Figure 1 — Effective vitamin A supplementation for prevention of BPD and mortality. (A) The forest plot for the incidence of oxygen dependency for 28 days among survivors. (B) The forest plot for the incidence of oxygen dependency at 36 weeks' PMA among survivors. (C) The forest plot for the incidence of death before 1 month. (D) The forest plot for the incidence of death at 36 weeks' PMA. [file Image_1.TIF]

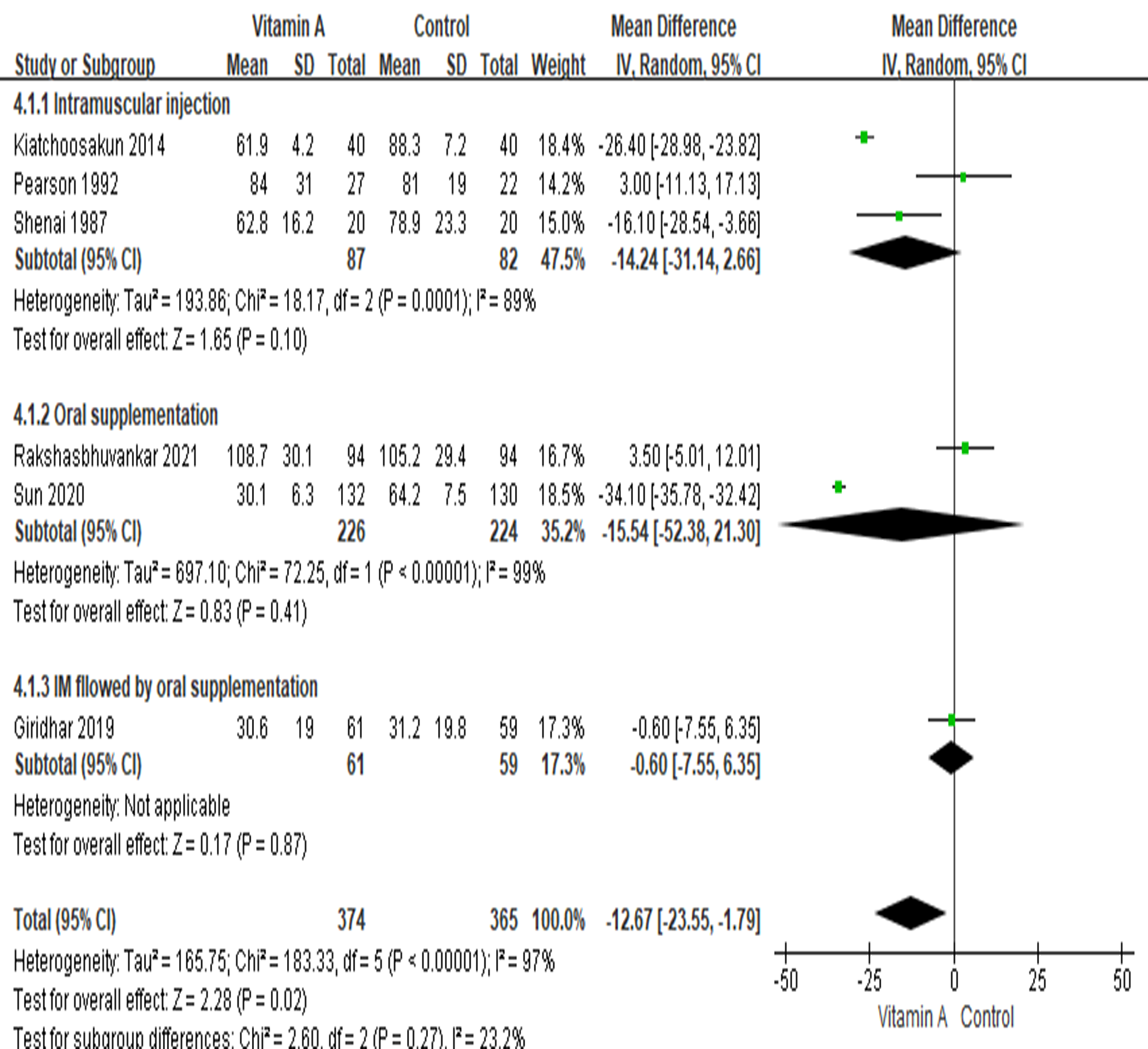

Supplement: Supplementary Figure 2 — The forest plot for the length of hospital stay. [file Image_2.TIF]

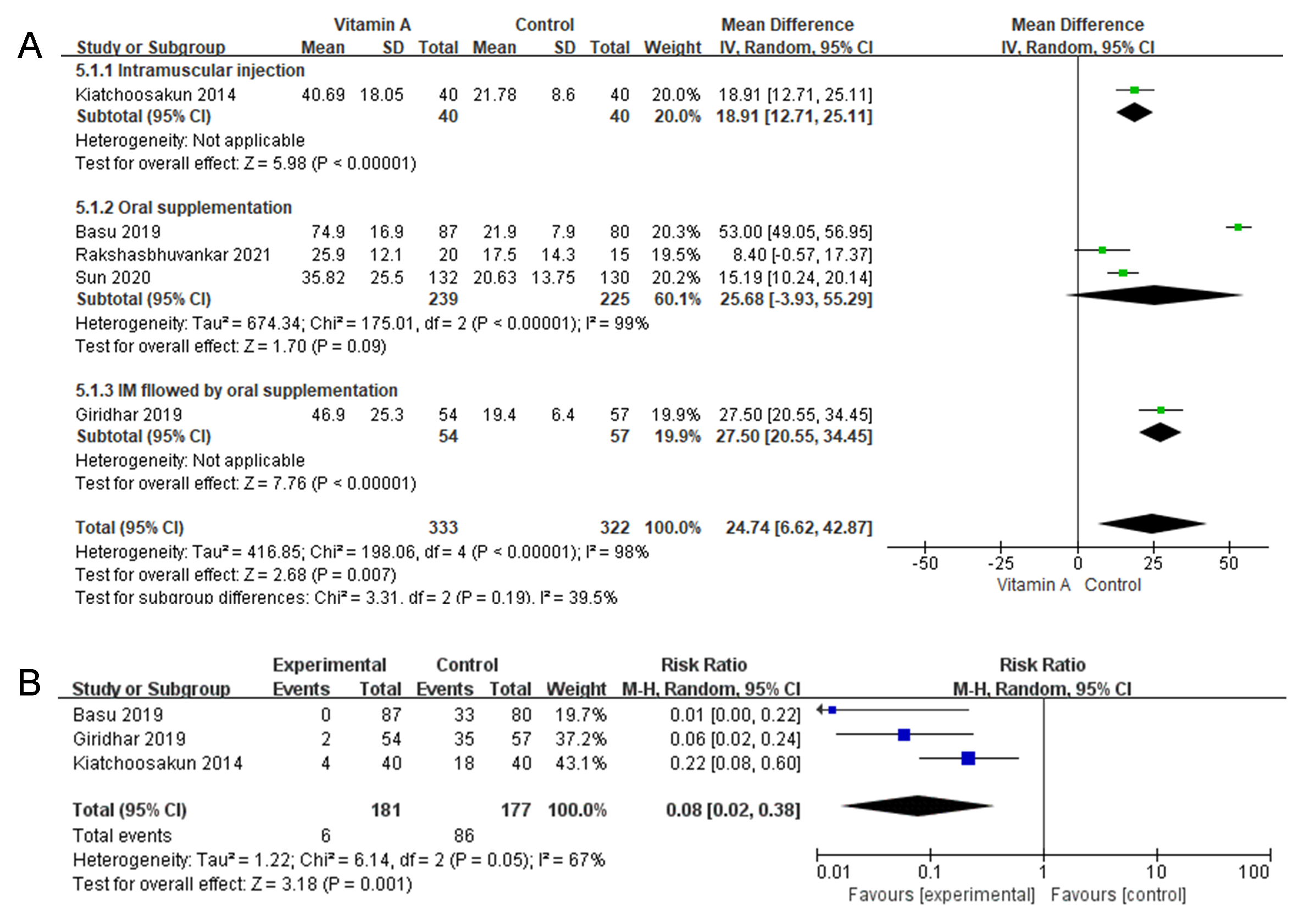

Supplement: Supplementary Figure 3 — (A) The forest plot for the plasma retinol. (B) The forest plot for the incidences of vitamin A deficiency. [file Image_3.TIF]

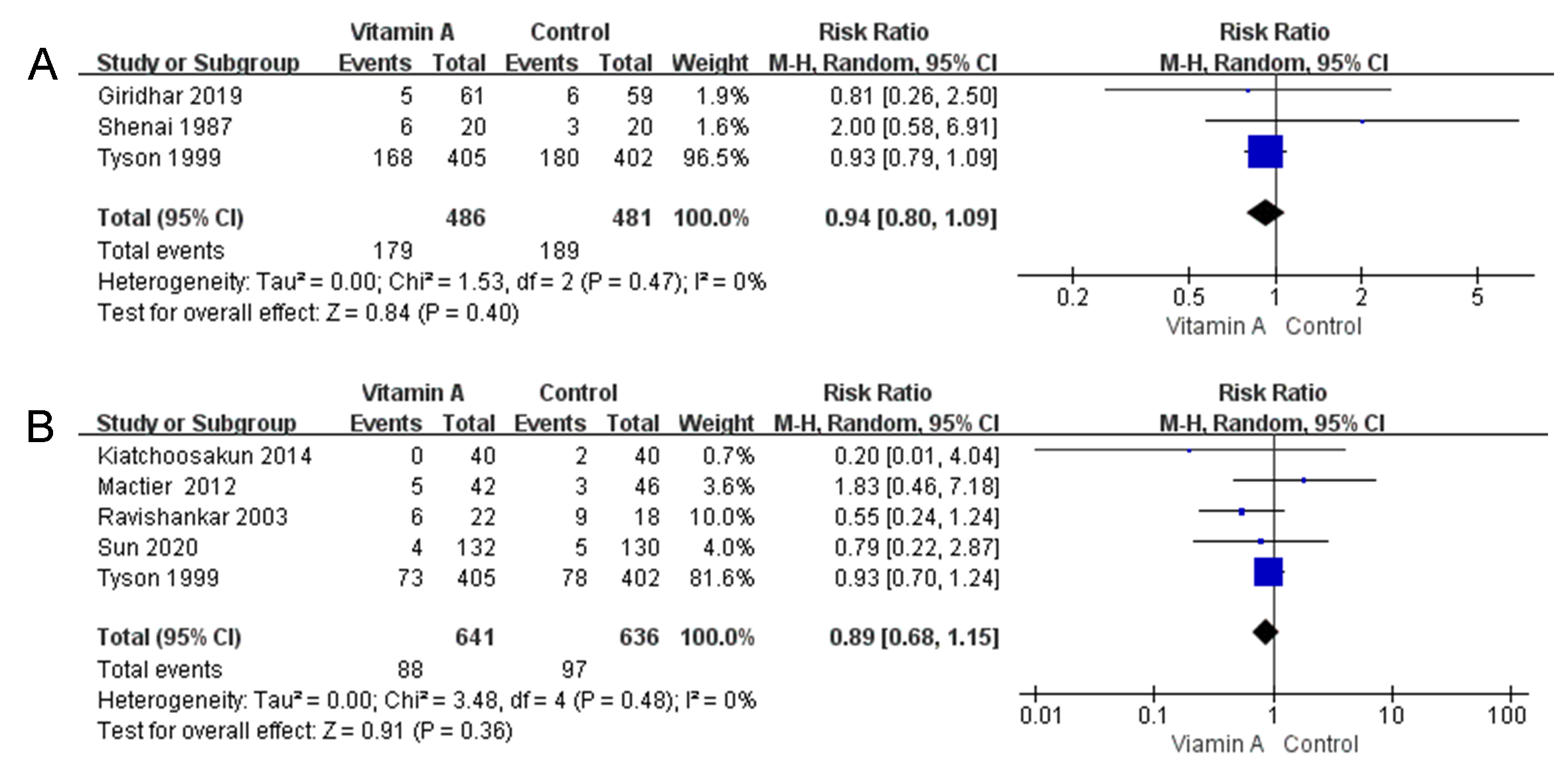

Supplement: Supplementary Figure 4 — The forest plot for the incidences of IVH. (A) The forest plot for the incidence of IVH of any grade. (B) The forest plot for the incidence of IVH of grade 3 or 4. [file Image_4.TIF]

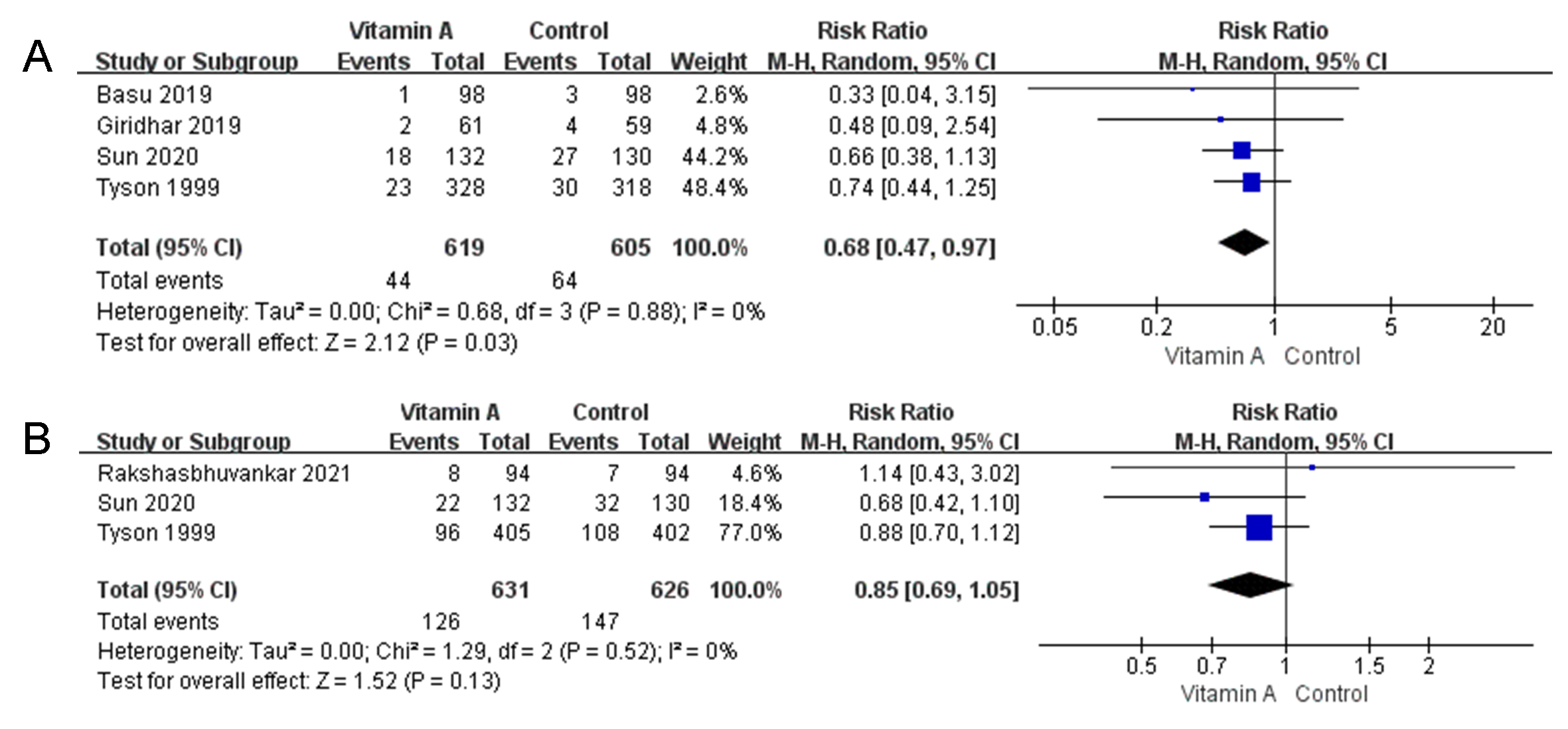

Supplement: Supplementary Figure 5 — The forest plot for the incidences of PVL. (A) The forest plot for the incidence of PVL. (B) The forest plot for the incidence of IVH of grade 3 or 4 or PVL. [file Image_5.TIF]

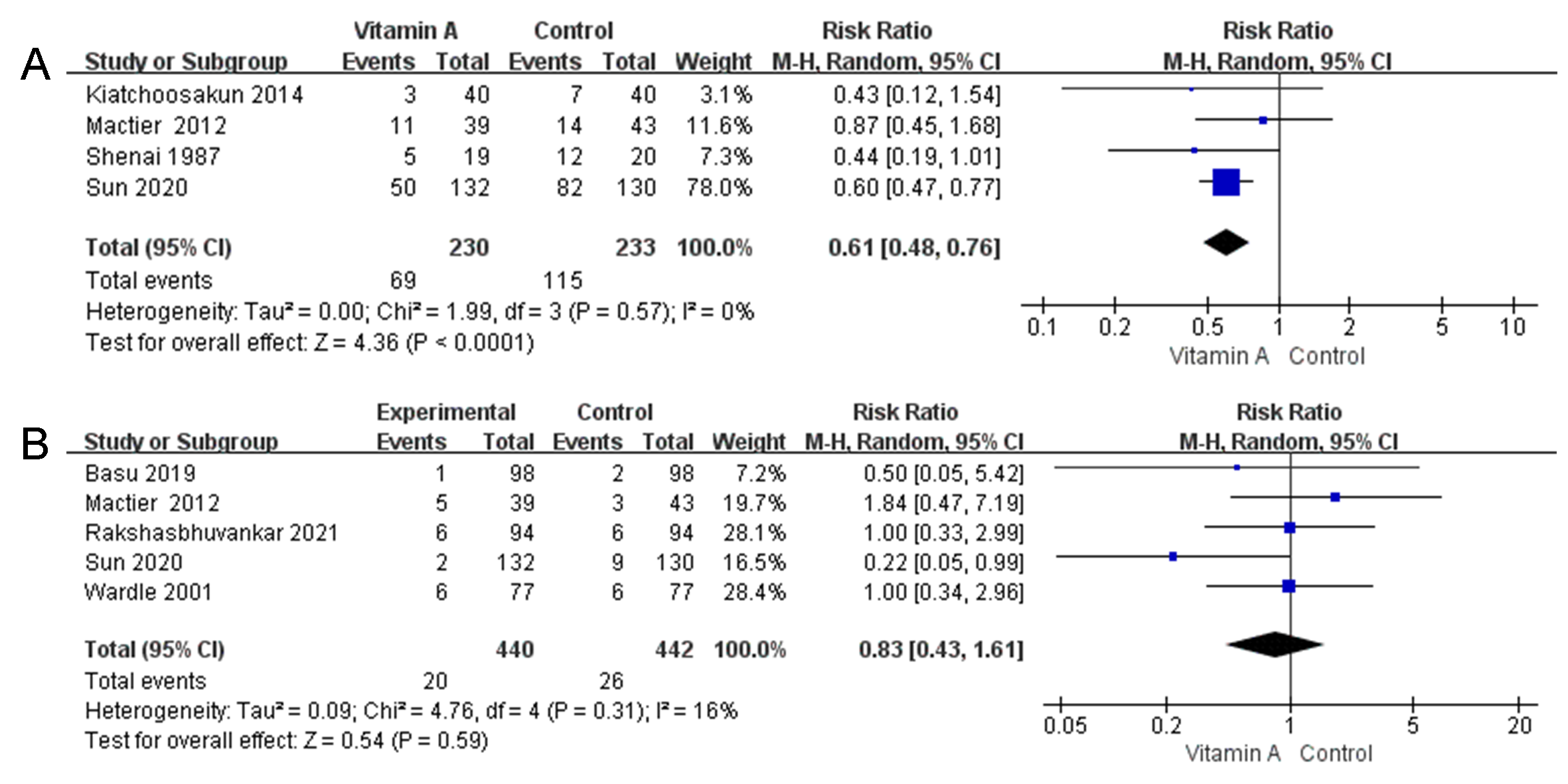

Supplement: Supplementary Figure 6 — The forest plot for the incidences of ROP. (A) The forest plot for the incidence of ROP of any grade. (B) The forest plot for the incidence of ROP requiring treatment. [file Image_6.TIF]

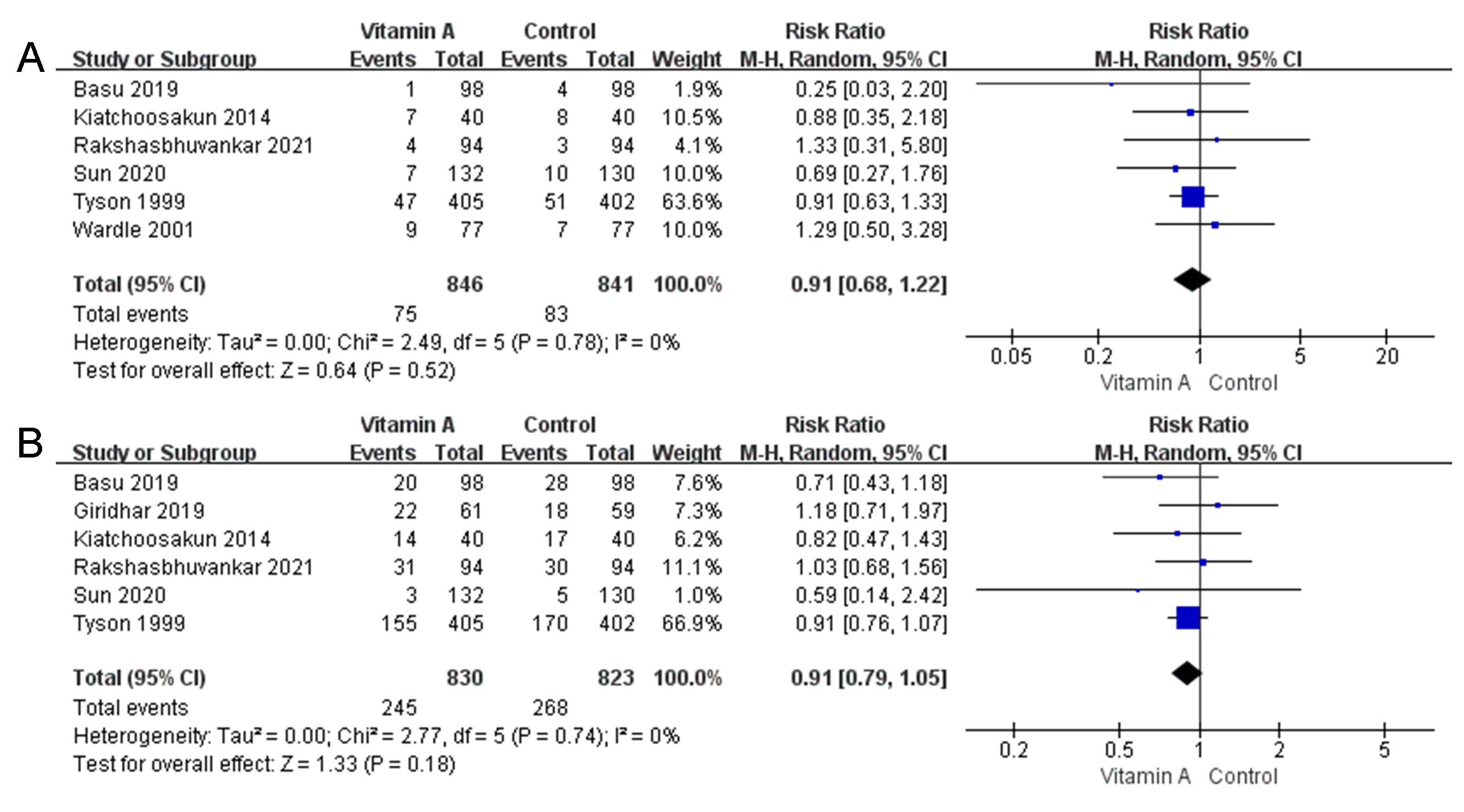

Supplement: Supplementary Figure 7 — (A) The forest plot for the incidence of NEC. (B) The forest plot for the incidence of sepsis. [file Image_7.TIF]
