# Supplementary material for: Early Vitamin A Supplementation for Prevention of Short-Term Morbidity and Mortality in Very-Low-Birth-Weight Infants: A Systematic Review and Meta-Analysis
Source: Front Pediatr. 2022 Apr 7;10:788409. doi: 10.3389/fped.2022.788409 (PMC9021759; doi:10.3389/fped.2022.788409)
Supplement: Supplementary 1 — Search strategies. [file Table_1.DOCX]

Search strategies

A: MEDLINE(via PubMed)

1 Infant, Very Low Birth Weight[MeSH Terms]/11,124

2 (((((Very-Low-Birth-Weight Infant[Title/Abstract]) OR (Infant, Very-Low-Birth-Weight[Title/Abstract])) OR (Infants, Very-Low-Birth-Weight[Title/Abstract])) OR (Very Low Birth Weight Infant[Title/Abstract])) OR (Very-Low-Birth-Weight Infants[Title/Abstract])) OR (Very Low Birth Weight[Title/Abstract])/8,079

3 ((((((Infant, Very Low Birth Weight[MeSH Terms]) OR (Very-Low-Birth-Weight Infant[Title/Abstract])) OR (Infant, Very-Low-Birth-Weight[Title/Abstract])) OR (Infants, Very-Low-Birth-Weight[Title/Abstract])) OR (Very Low Birth Weight Infant[Title/Abstract])) OR (Very-Low-Birth-Weight Infants[Title/Abstract])) OR (Very Low Birth Weight[Title/Abstract])/14,717

4 vitamin A[MeSH Terms]/46,042

5 (((((Aquasol A[Title/Abstract]) OR (Retinol[Title/Abstract])) OR (All-Trans-Retinol[Title/Abstract])) OR (All Trans Retinol[Title/Abstract])) OR (Vitamin A1[Title/Abstract])) OR (11-cis-Retinol[Title/Abstract])/14,716

6 (vitamin A[MeSH Terms]) OR ((((((Aquasol A[Title/Abstract]) OR (Retinol[Title/Abstract])) OR (All-Trans-Retinol[Title/Abstract])) OR (All Trans Retinol[Title/Abstract])) OR (Vitamin A1[Title/Abstract])) OR (11-cis-Retinol[Title/Abstract]))/53,305

7 (randomized controlled trial[Publication Type]) OR (controlled clinical trial[Publication Type])/647,277

8 (((((randomized[Title/Abstract]) OR (placebo[Title/Abstract])) OR (randomly[Title/Abstract])) OR (trial[Title/Abstract])) OR (groups[Title/Abstract])) OR (drug therapy[MeSH Subheading])/5,213,366

9 ((randomized controlled trial[Publication Type]) OR (controlled clinical trial[Publication Type])) OR ((((((randomized[Title/Abstract]) OR (placebo[Title/Abstract])) OR (randomly[Title/Abstract])) OR (trial[Title/Abstract])) OR (groups[Title/Abstract])) OR (drug therapy[MeSH Subheading]))/5,309,485

10 ((((((((Very-Low-Birth-Weight Infant[Title/Abstract]) OR (Infant, Very-Low-Birth-Weight[Title/Abstract])) OR (Infants, Very-Low-Birth-Weight[Title/Abstract])) OR (Very Low Birth Weight Infant[Title/Abstract])) OR (Very-Low-Birth-Weight Infants[Title/Abstract])) OR (Very Low Birth Weight[Title/Abstract])) OR (Infant, Very Low Birth Weight[MeSH Terms])) AND ((vitamin A[MeSH Terms]) OR ((((((Aquasol A[Title/Abstract]) OR (Retinol[Title/Abstract])) OR (All-Trans-Retinol[Title/Abstract])) OR (All Trans Retinol[Title/Abstract])) OR (Vitamin A1[Title/Abstract])) OR (11-cis-Retinol[Title/Abstract])))) AND (((randomized controlled trial[Publication Type]) OR (controlled clinical trial[Publication Type])) OR ((((((randomized[Title/Abstract]) OR (placebo[Title/Abstract])) OR (randomly[Title/Abstract])) OR (trial[Title/Abstract])) OR (groups[Title/Abstract])) OR (drug therapy[MeSH Subheading])))/56

B: Embase (via OvidSP) 1974 to 2022 January 1

1 exp very low birth weight/ (16314)

2 (birth weight, very low or infant, very low birth weight or very low birth weight infant or very low birthweight or VLBW).ab. (6832)

3 1 or 2 (17557)

4 exp retinol/ (41937)

5 (vitamin A or retinol or retinoid or retinoic or aquasol).ab. (75403)

6 4 or 5 (96173)

7 (randomized controlled trial or controlled clinical trial).pt. or randmoized.ab. or placbo.ab. or drug therapy.ab. or randomly.ab. or trial.ab. or groups.ab. (4077121)

8 3 and 6 and 7 (61)

C: CENTRAL

#1 MeSH descriptor: [Infant, Very Low Birth Weight] explode all trees 1014

#2 (Very-Low-Birth-Weight Infant):ti,ab,kw OR (Infant, Very-Low-Birth-Weight):ti,ab,kw OR (Infants, Very-Low-Birth-Weight):ti,ab,kw OR (Very Low Birth Weight Infant):ti,ab,kw OR (Very-Low-Birth-Weight Infants or Very Low Birth Weight):ti,ab,kw (Word variations have been searched) 2777

#3 #1 or #2 2872

#4 MeSH descriptor: [Vitamin A] explode all trees 2127

#5 (vitamin A):ti,ab,kw OR (retinol):ti,ab,kw OR (retinoid):ti,ab,kw OR (retinoic):ti,ab,kw OR (aquasol):ti,ab,kw 29671

#6 #4 or #5 30006

#7 #3 and #6 in Trials RCTS only 11

D：Web of Science

#4 122 #3  AND  #2  AND  #1

Indexes=SCI-EXPANDED, SSCI, A&HCI, CPCI-S, CPCI-SSH, ESCI, CCR-EXPANDED, IC Timespan=All years

#3 2,213,961

TS=(randomized  controlled  trial  or  random)

Indexes=SCI-EXPANDED, SSCI, A&HCI, CPCI-S, CPCI-SSH, ESCI, CCR-EXPANDED, IC Timespan=All years

#2 577,780 TS=(Vitamin  A  or  Aquasol  A  or  Retinol  or  3,7-dimethyl-9-(2,6,6-trimethyl-1-cyclohexen-1-yl)-2,4,6,8-nonatetraen-1-ol,(all-E)-Isomer  or  All-Trans-Retinol  or  All  Trans  Retinol  or  Vitamin  A1  or  11-cis-Retinol)

Indexes=SCI-EXPANDED, SSCI, A&HCI, CPCI-S, CPCI-SSH, ESCI, CCR-EXPANDED, IC Timespan=All years

#1 24,894

TS=(very low birth weight or birth weight, very low or infant, very low birth weight or very low birth weight infant or very low birthweight or VLBW )

Indexes=SCI-EXPANDED, SSCI, A&HCI, CPCI-S, CPCI-SSH, ESCI, CCR-EXPANDED, IC Timespan=All years
