# Supplementary material for: Early Vitamin A Supplementation for Prevention of Short-Term Morbidity and Mortality in Very-Low-Birth-Weight Infants: A Systematic Review and Meta-Analysis
Source: Front Pediatr. 2022 Apr 7;10:788409. doi: 10.3389/fped.2022.788409 (PMC9021759; doi:10.3389/fped.2022.788409)
Supplement: Supplementary 2 — Risk of bias assessment. [file Table_2.DOCX]

**Supplementary 2**. Risk of bias assessment

1. Risk of bias table of **Basu 2019**

| Bias | Author’s judgement | Support for judgement |
| --- | --- | --- |
| Random sequence generation (selection bias) | Low risk | “ Randomization into vitamin A or placebo group was done using random permuted blocks of 4, 6, and 8, prepared by an independent statistician not involved in the study.” |
| Allocation concealment (selection bias) | Low risk | “ Allocation into vitamin A or placebo group was done using serially numbered opaque and sealed envelopes by on-duty residents who were appropriately trained for the process beforehand. Allocation concealment was maintained throughout  the study.” |
| Blinding of participants and personnel (performance bias) | Low risk | “ Treating physicians, nursing staffs, and the parents were unaware about the composition of the bottles.” |
| Blinding of outcome assessment (detection bias) | Unclear risk | Blinding of outcome assessment was not described. |
| Incomplete outcome data (attrition bias) | Low risk | The numbers and reasons for dropouts and withdrawals in all groups were described. |
| Selective reporting (reporting bias) | Low risk | Pre-defined and clinically relevant outcomes were reported. |
| Other bias | Unclear risk | Source of funding was not reported. |

2.Risk of bias table of **Giridhar 2019**

| Bias | Author’s judgement | Support for judgement |
| --- | --- | --- |
| Random sequence generation (selection bias) | Low risk | “The random sequence was generated online from the website [www.randomizer.org](http://www.randomizer.org).” |
| Allocation concealment (selection bias) | Unclear risk | No information provided regarding allocation concealment. |
| Blinding of participants and personnel (performance bias) | Low risk | “The investigators, supervisors, caregivers, laboratory personnel, and statistician were blinded to the intervention.” |
| Blinding of outcome assessment (detection bias) | Low risk | “The investigators, supervisors, caregivers, laboratory personnel, and statistician were blinded to the intervention. The randomization sequence code was opened only after completion of biochemical and statistical analysis.” |
| Incomplete outcome data (attrition bias) | Low risk | The numbers and reasons for dropouts and withdrawals in all groups were described. |
| Selective reporting (reporting bias) | Low risk | Pre-defined and clinically relevant outcomes were reported. |
| Other bias | Unclear risk | Source of funding was not reported. |

3. Risk of bias table of **Kiatchoosakun 2014**

| Bias | Author’s judgement | Support for judgement |
| --- | --- | --- |
| Random sequence generation (selection bias) | Low risk | “The infants were assigned to a vitamin A or control group by a research nurse using a randomization list (with sealed envelopes containing the treatment assignments randomized by blocks of 4).” |
| Allocation concealment (selection bias) | Low risk | “The infants were assigned to a vitamin A or control group by a research nurse using a randomization list (with sealed envelopes containing the treatment assignments randomized by blocks of 4).” |
| Blinding of participants and personnel (performance bias) | Low risk | “A screen was placed around the bed, a pacifier was used for non-pharmacological pain management, and the injection site was covered with cotton and tape. The same covering was placed on the control infants. The research nurse removed the covering at the next treatment. (Owing to the small needle, the injection site was not visible to the nurse).” |
| Blinding of outcome assessment (detection bias) | Unclear risk | Blinding of outcome assessment was not described. |
| Incomplete outcome data (attrition bias) | Low risk | There are no dropouts and all the infants were accounted for. |
| Selective reporting (reporting bias) | Low risk | Pre-defined and clinically relevant outcomes were reported. |
| Other bias | Unclear risk | Source of funding was not reported. |

4. Risk of bias table of **Mactier 2012**

| Bias | Author’s judgement | Support for judgement |
| --- | --- | --- |
| Random sequence generation (selection bias) | Low risk | “Randomization was undertaken by pharmacy staff, who had no contact with study participants, and was stratified for gestational age and hospital of birth using a comminuted block method.” |
| Allocation concealment (selection bias) | Low risk | “Following randomization, sealed bags labeled with the infant’s details and containing either an empty box or a vial of Aquasol A within a similar box were delivered to the neonatal unit. Bags were opened by attending nursing staff without the presence of the research nurse and vitamin A given as appropriate.” |
| Blinding of participants and personnel (performance bias) | Low risk | The mock injection was used. |
| Blinding of outcome assessment (detection bias) | Low risk | “ The entire research team remained blinded to each infant’s randomization until all ERG data had been analyzed.” |
| Incomplete outcome data (attrition bias) | Low risk | The numbers and reasons for dropouts and withdrawals in all groups were described. |
| Selective reporting (reporting bias) | Low risk | Clinically relevant outcomes were reported. |
| Other bias | Low risk |  |

5. Risk of bias table of **Pearson 1992**

| Bias | Author’s judgement | Support for judgement |
| --- | --- | --- |
| Random sequence generation (selection bias) | Low risk | “Randomization was performed with the use of  sequential, sealed envelopes.” |
| Allocation concealment (selection bias) | Low risk | “Randomization was performed with the use of  sequential, sealed envelopes.” |
| Blinding of participants and personnel (performance bias) | Low risk | “A similar volume of 0.9% saline solution was used as a placebo. Opaque tape concealed the contents of syringes to ensure blinding.”  “At all three sites, staff responsible for patient care had no knowledge of group designation.” |
| Blinding of outcome assessment (detection bias) | Unclear risk | Blinding of outcome assessment was not described. |
| Incomplete outcome data (attrition bias) | Low risk | All the infants were accounted for. |
| Selective reporting (reporting bias) | Low risk | Clinically relevant outcomes were reported. |
| Other bias | Unclear risk | “The estimated sample size was 41 subjects per  treatment group.” The study enrolled 49 infants (27/22). |

6.Risk of bias table of **Rakshasbhuvankar 2021**

| Bias | Author’s judgement | Support for judgement |
| --- | --- | --- |
| Random sequence generation (selection bias) | Low risk | “Infants were randomly assigned by a hospital pharmacist not involved in clinical care. Randomization followed a computer-generated randomization table using blocks of 6.” |
| Allocation concealment (selection bias) | Low risk | “Randomization was performed within the Research Electronic Data Capture randomization module, ensuring allocation concealment throughout the study period.” |
| Blinding of participants and personnel (performance bias) | Low risk | “The pharmacy dispensed vitamin A and the placebo in identical amber-colored containers. Both preparations were indistinguishable by their appearance, smell, and other physical properties.” |
| Blinding of outcome assessment (detection bias) | Low risk | “Deidentified data were stored in a secure, Web-based, and password-protected Research Electronic Data Capture system.” |
| Incomplete outcome data (attrition bias) | Low risk | The numbers and reasons for dropouts and withdrawals in all groups were described. |
| Selective reporting (reporting bias) | Low risk | Pre-defined and clinically relevant outcomes were reported. |
| Other bias | Low risk |  |

7. Risk of bias table of **Ravishanar 2003**

| Bias | Author’s judgement | Support for judgement |
| --- | --- | --- |
| Random sequence generation (selection bias) | Low risk | “The infants were randomly assigned to one of two  groups. Sealed envelopes containing cards with the group designation were maintained in the pharmacy department.” |
| Allocation concealment (selection bias) | Low risk | “ Sealed envelopes containing cards with the group designation were maintained in the pharmacy department.” |
| Blinding of participants and personnel (performance bias) | Low risk | “ The staff responsible for the care of these infants had no knowledge of group assignment or outcome of study echocardiograms.” |
| Blinding of outcome assessment (detection bias) | Low risk | “ One of the authors who was blinded to group assignment performed all of the analyses.” |
| Incomplete outcome data (attrition bias) | Low risk | There are no dropouts and all the infants were accounted for. |
| Selective reporting (reporting bias) | Unclear risk | A limited number clinically relevant outcomes were reported, not all expected outcomes were reported. |
| Other bias | Unclear risk | Source of funding was not reported. |

8. Risk of bias table of **Shenai 1987**

| Bias | Author’s judgement | Support for judgement |
| --- | --- | --- |
| Random sequence generation (selection bias) | Low risk | “Sealed envelopes containing cards indicating group designation were used for randomization. The randomization was carried out by pharmacists who had no knowledge of the clinical status of the infants. ” |
| Allocation concealment (selection bias) | Low risk | “The randomization was carried out by pharmacists who had no knowledge of the clinical status of the infants.” |
| Blinding of participants and personnel (performance bias) | Low risk | “The neonatal intensive care unit staff responsible for patient management had no knowledge of the group designation.” |
| Blinding of outcome assessment (detection bias) | Unclear risk | Blinding of outcome assessment was not described. |
| Incomplete outcome data (attrition bias) | Low risk | All the infants were accounted for. |
| Selective reporting (reporting bias) | Low risk | Clinically relevant outcomes were reported. |
| Other bias | Low risk |  |

9. Risk of bias table of **Sun 2020**

| Bias | Author’s judgement | Support for judgement |
| --- | --- | --- |
| Random sequence generation (selection bias) | Low risk | “ A blocked randomization method stratified by the  neonatal intensive care unit size was used to assign  infants to either the control or oral VA group.” |
| Allocation concealment (selection bias) | Low risk | “The medical and nursing teams caring for the infants were thus completely unaware of the content of the solutions, which were only labeled with the study site and infant number.” |
| Blinding of participants and personnel (performance bias) | Low risk | “ An equivalent volume of the placebo solution was provided in the same way in the control group. The placebo solution was prepared with soybean oil by the hospital pharmacist having the same aspect as the VA solution. The medical and nursing teams caring for the infants were thus completely unaware of the content of the solutions, which were only labeled with the study site and infant number.” |
| Blinding of outcome assessment (detection bias) | Unclear risk | Blinding of outcome assessment was not described. |
| Incomplete outcome data (attrition bias) | Low risk | The numbers and reasons for dropouts and withdrawals in all groups were described. |
| Selective reporting (reporting bias) | Low risk | Pre-defined and clinically relevant outcomes were reported. |
| Other bias | Unclear risk | Sample size calculation was not reported. |

10. Risk of bias table of **Tyson 1999**

| Bias | Author’s judgement | Support for judgement |
| --- | --- | --- |
| Random sequence generation (selection bias) | Low risk | “The infants were stratified according to center and birth weight and assigned to the vitamin A or control group by a hospital pharmacist using a randomization list (or, at four centers, by a research nurse using sealed envelopes containing the treatment assignments).” |
| Allocation concealment (selection bias) | Low risk | “ The infants were assigned to the vitamin A or control group by a hospital pharmacist using a randomization list (or, at four centers, by a research nurse using sealed envelopes containing the treatment assignments).” |
| Blinding of participants and personnel (performance bias) | Low risk | “ Control infants received a sham procedure  rather than placebo injections.”  “ With the small needle, the injection site was either not visible or visible only on close inspection.” |
| Blinding of outcome assessment (detection bias) | Low risk | “ An independent data safety and monitoring committee used the Lan–DeMets procedure with an O’Brien–Fleming spending function to assess the intervention.” |
| Incomplete outcome data (attrition bias) | Low risk | All infants were accounted for. |
| Selective reporting (reporting bias) | Low risk | Clinically relevant outcomes were reported. |
| Other bias | Low risk |  |

11. Risk of bias table of **Wardle 2001**

| Bias | Author’s judgement | Support for judgement |
| --- | --- | --- |
| Random sequence generation (selection bias) | Low risk | “ Infants were randomised using sealed opaque  numbered envelopes containing the treatment  allocation, which had been assigned using a  computerised random number generator.” |
| Allocation concealment (selection bias) | Low risk | “ Infants were randomised using sealed opaque  numbered envelopes containing the treatment  allocation.” |
| Blinding of participants and personnel (performance bias) | Low risk | “ Both the medical and nursing staV caring for  the infants and administering the vitamin A  and placebo solutions were unaware of the  treatment allocation.” |
| Blinding of outcome assessment (detection bias) | Low risk | “ The identity of the treatments was not available to the authors until after the data had been analysed.” |
| Incomplete outcome data (attrition bias) | Low risk | All the infants were accounted for. |
| Selective reporting (reporting bias) | Low risk | Clinically relevant outcomes were reported. |
| Other bias | Low risk |  |

12. Risk of bias table of **Werkman 1994**

| Bias | Author’s judgement | Support for judgement |
| --- | --- | --- |
| Random sequence generation (selection bias) | Unclear risk | “Eligible infants were enrolled and were randomly assigned to one of two vitamin A treatments.”  The method of sequence generation was not reported. |
| Allocation concealment (selection bias) | Unclear risk | No information provided regarding allocation concealment. |
| Blinding of participants and personnel (performance bias) | Unclear risk | It is not mentioned if the trial was blinded |
| Blinding of outcome assessment (detection bias) | High risk | There was no blinding in outcome assessment. |
| Incomplete outcome data (attrition bias) | High risk | “Additional infants were enrolled but were transferred to another hospital for surgery (n = 10) or died from sepsis (n = 2). Their data were not included.” |
| Selective reporting (reporting bias) | Unclear risk | A limited number clinically relevant outcomes were reported, not all expected outcomes were reported. |
| Other bias | High risk | There were methodological problems. |
